# Etiology, Risk Factors, and Diagnosis of Back Pain in Children and Adolescents: Evidence- and Consensus-Based Interdisciplinary Recommendations

**DOI:** 10.3390/children9020192

**Published:** 2022-02-02

**Authors:** Michael Frosch, Maximilian D. Mauritz, Stefan Bielack, Susanne Blödt, Uta Dirksen, Michael Dobe, Florian Geiger, Renate Häfner, Lea Höfel, Bettina Hübner-Möhler, Thekla von Kalle, Burkhard Lawrenz, Andreas Leutner, Frauke Mecher, Kiril Mladenov, Heike Norda, Lorin Stahlschmidt, Marc Steinborn, Ralf Stücker, Ralf Trauzeddel, Regina Trollmann, Julia Wager, Boris Zernikow

**Affiliations:** 1 German Paediatric Pain Centre, Children′s and Adolescents′ Hospital, 45711 Datteln, Germany; m.mauritz@kinderklinik-datteln.de (M.D.M.); m.dobe@kinderklinik-datteln.de (M.D.); b.huebner-moehler@deutsches-kinderschmerzzentrum.de (B.H.-M.); l.stahlschmidt@deutsches-kinderschmerzzentrum.de (L.S.); j.wager@deutsches-kinderschmerzzentrum.de (J.W.); b.zernikow@kinderklinik-datteln.de (B.Z.); 2 Department of Children’s Pain Therapy and Paediatric Palliative Care, Faculty of Health, School of Medicine, Witten/Herdecke University, 58448 Witten, Germany; 3 Klinikum Stuttgart, Olgahospital, Stuttgart Cancer Center, Center for Pediatric, Adolescent and Women’s Medicine, Pediatrics 5 (Oncology, Hematology, Immunology), 70174 Stuttgart, Germany; S.Bielack@klinikum-stuttgart.de; 4 Arbeitsgemeinschaft der Wissenschaftlichen Medizinischen Fachgesellschaften—Institut für Medizinisches Wissensmanagement (AWMF-IMWI), Philipps-Universität Marburg, 35043 Marburg, Germany; bloedt@awmf.org; 5 Pediatrics III, West German Cancer Center, University Hospital Essen, 45147 Essen, Germany; Uta.Dirksen@uk-essen.de; 6 Spine Center, Hessing Foundation, 86199 Augsburg, Germany; Florian.Geiger@hessing-stiftung.de; 7 German Center for Pediatric and Adolescent Rheumatology and Center for Pain Therapy for Young People, 82467 Garmisch-Partenkirchen, Germany; haefner.renate@rheuma-kinderklinik.de (R.H.); hoefel.lea@rheuma-kinderklinik.de (L.H.); 8 Department of Pediatric Radiology, Olgahospital, Klinikum Stuttgart, 70174 Stuttgart, Germany; T.vonKalle@klinikum-stuttgart.de; 9 Private Practice for Paediatrics and Adolescent Medicine, 59821 Arnsberg, Germany; blawrenz@t-online.de; 10 Department of Pediatric Surgery, Medical Center Dortmund, 44137 Dortmund, Germany; Andreas.Leutner@klinikumdo.de; 11 Physio Deutschland, German Federal Association for Physiotherapy, 50679 Cologne, Germany; Info@physio-deutschland.de; 12 Department of Pediatric Orthopaedic Surgery, Children’s Hospital Hamburg-Altona, University Medical Center Hamburg-Eppendorf, 22763 Hamburg, Germany; kiril.mladenov@kinderkrankenhaus.net (K.M.); ralf.stuecker@kinderkrankenhaus.net (R.S.); 13 SchmerzLOS e.V. (Patient Advocacy Group), 23556 Lubeck, Germany; norda@schmerzlos-ev.de; 14 Department of Diagnostic and Interventional and Pediatric Radiology, München Klinik Schwabing, 80804 Munich, Germany; marc.steinborn@klinikum-muenchen.de; 15 Department of Pediatrics, Pediatric and Adolescent Rheumatology, Helios Klinik Berlin-Buch, 13125 Berlin, Germany; ralf.trauzeddel@helios-gesundheit.de; 16 Department of Pediatrics, Division of Neuropaediatrics, Friedrich-Alexander-Universität Erlangen-Nürnberg, 91054 Erlangen, Germany; regina.trollmann@uk-erlangen.de

**Keywords:** guideline, back pain, etiology, risk factors, diagnosis, children, adolescents, evidence-based

## Abstract

Using a structured approach and expert consensus, we developed an evidence-based guideline on the diagnosis of back pain and the treatment of non-specific back pain in children and adolescents. The first part comprises etiology, risk factors, and diagnosis. The second part, published in the same issue, includes treatment and prevention. A comprehensive and systematic literature search was conducted to identify relevant guidelines and studies. Based on the findings of this literature search, recommendations on risk factors and diagnosis were formulated and voted on by experts in a structured consensus-building process. Notable red flags for specific back pain and evidence-based risk factors for non-specific back pain in children and adolescents were identified. Only three evidence-based recommendations could be formulated for causes, red flags, and risk factors for back pain, while two recommendations are based on expert consensus. Regarding diagnostics, eight expert consensus recommendations and one evidence-based recommendation could be provided. Despite the importance of adequate diagnosis for the treatment of back pain in children and adolescents, results of this work confirm the deficit in research investment in this area.

## 1. Introduction

Back pain is a relevant and increasing problem in children’s and adolescents’ health [1]. Back pain can occur with an underlying disease (i.e., specific back pain) or without evident cause of the complaint (i.e., non-specific back pain). For specific back pain in childhood and adolescence, an extensive spectrum of differential diagnoses, including a wide variety of congenital and acquired underlying diseases, must be considered as potentially causative of the pain [1]. Non-specific back pain represents the largest group of back pain in adolescence [2] and is a predictor of chronic back pain in adults [3]. While it is relatively rare in childhood, the prevalence of non-specific back pain rapidly increases in adolescence [4]. Early and reliable differentiation between specific and non-specific back pain is crucial for targeted therapy.

A few diagnostic algorithms have been proposed to detect specific causes of back pain in children and adolescents (e.g., [5,6]). The difficultly with such algorithms is balancing the risks versus the benefits of different diagnostic measures [5]. Various red flags and algorithms have been published to support the diagnostics of back pain in childhood and adolescence [7,8,9]. However, these are often not evidence-based but have been developed by clinical experts [5,10]. Evidence-based guidelines on the diagnosis and treatment of non-specific back pain in childhood and adolescence are lacking. Therefore, we developed a guideline based both on the best available evidence sourced with a systematic literature search and on expert consensus with a working group that included all relevant local expert institutions.

This guideline is intended to provide the basis for age-specific optimization of diagnosis and treatment of back pain in children and adolescents. The guideline applies to pediatric patients whose leading symptom is back pain. Both specific and non-specific back pain are considered. The diagnostics for the detection of specific causes also concerns acute diseases, such as infections or injury-associated back pain. Therefore, the guideline includes differential diagnosis in the early course of back pain in childhood and adolescence. It targets physicians of various specialties (particularly pediatrics, pediatric surgery, orthopedics, and radiology), physical therapists and psychotherapists, as well as patients and parents. The guideline serves as an orientation for individual diagnostic and therapeutic decisions in primary and specialized care of children and adolescents.

The first part of this guideline focuses on the etiology, risk factors, and diagnosis of back pain in children and adolescents. The second part (see Frosch et al. in this special issue) describes therapy and prevention of non-specific back pain in children and adolescents.

## 2. Materials and Methods

This guideline was prepared on behalf of the German Society for Pediatric and Adolescent Medicine and developed according to the regulations of the Arbeitsgemeinschaft der Wissenschaftlichen Medizinischen Fachgesellschaften (AWMF (Working group of the German Scientific Medical Societies); [11]). A total of 14 professional societies, organizations and patient representatives agreed to participate in the development of the guideline. The professional societies were selected according to the regulations of the AWMF [11] and the topic of the guideline. Relevant societies and organizations were invited to participate. Additionally, the guideline was publicly announced so that further societies and organizations could contact and join the guideline working group. Each participating society or organization nominated one or two representatives with special expertise for the guideline topic. In a constituting meeting held in April 2018, the objectives, research questions, methodical approach to the systematic literature search, and the appraisal of evidence were defined. Working groups for the various topics were established.

Details of the systematic literature search, evidence assessment, and the external review are described in the second part of this guideline (see Frosch et al. in this special issue).

**Figure 1:**
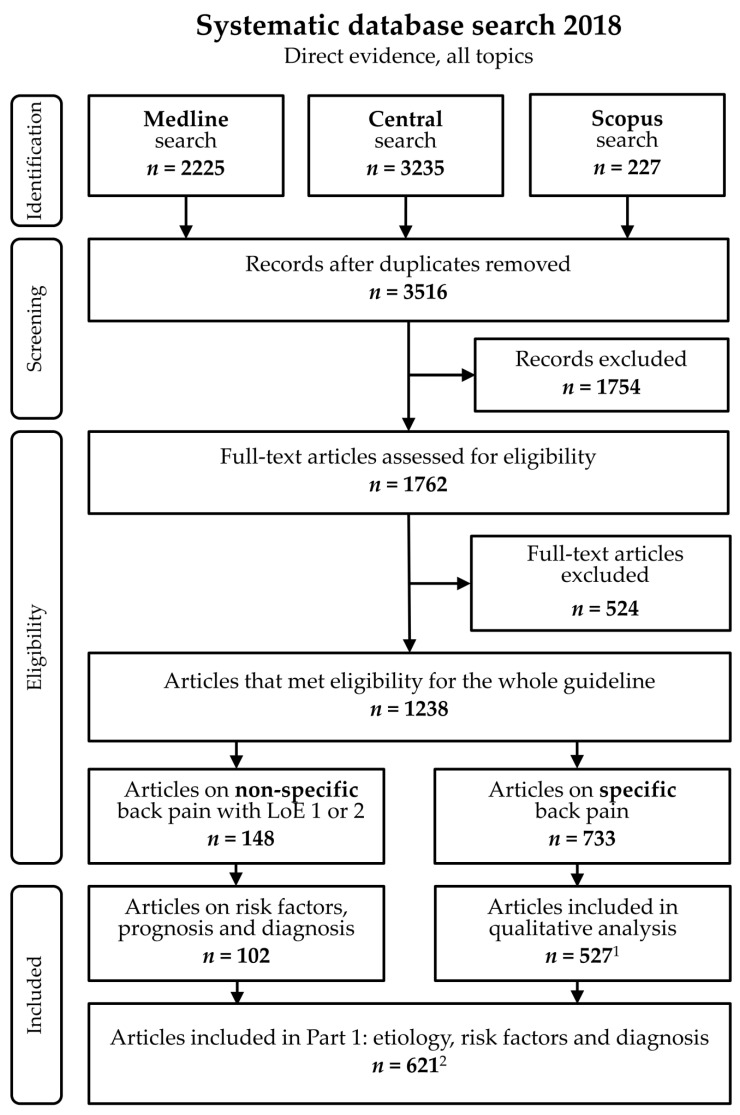
Flowchart. LoE = Level of evidence. ^1^ *n* = 206 articles did not contain sufficient information regarding underlying disease or concomitant symptoms for further analysis. ^2^ Eight articles were included for specific and non-specific back pain.

In summary, a systematic literature search was conducted in Medline, Central, and Scopus. Inclusion criteria were type of article (original article, systematic review, meta-analysis), language (English, German), and presence of back pain in children and adolescents between the ages of 3 and 18 years. Both studies on specific and non-specific back pain conditions were included. However, studies on specific back pain were only included for differential diagnostics to identify red flags. To systematically collect the diseases for which back pain is reported as a symptom, all studies on specific back pain were included, regardless of level of evidence. From these studies, relevant data were extracted concerning underlying diseases, medical history, attendant symptoms, and clinical findings. For all other topics, only studies with a level of evidence of 1 or 2 were included (according to the Oxford 2011 Levels of Evidence; [12]). For included studies, relevant data were extracted according to the regulations of the AWMF [11]. Figure 1 displays the results of the systematic literature searches.

Based on the systematic data extraction of the literature, the working groups developed statements and recommendations on each topic. These statements and recommendations were discussed and approved by all members of the guideline working group in a Delphi exercise in March 2021 and in a final consensus conference in April 2021. In the Delphi round, participants of the guideline working group could approve a statement/recommendation or propose an amendment. In the final online consensus conference, statements and recommendations that were not fully approved with 100% consensus in the Delphi round were discussed, reformulated if necessary, and again voted on. In both the Delphi exercise and the consensus conference, each participating society or organization could only vote once, even if there were two representatives.

All members of the guideline working group reported potential conflicts of interest. In cases where a conflict of interest was evident, the respective member abstained from voting on the relevant topic.

Based on the regulations of the AWMF [11], the strength of a recommendation was classified as “strong recommendation” (A), “recommendation” (B) or “open recommendation” (0). In the case of missing evidence, recommendations were based on expert consensus.

## 3. Results

A total of *n* = 3516 articles were screened for inclusion, with *n* = 621 individual articles included for analysis. Fourteen recommendations on red flags, risk factors, and diagnostics of back pain in children and adolescents were edited and endorsed by pediatric surgery, neurology, oncology, radiology, rheumatology, orthopedics, pain medicine, psychology, and physical therapy specialists.

### 3.1. Causes, Red Flags and Risk Factors

#### 3.1.1. Causes and Red Flags for Specific back Pain in Childhood and Adolescence

The literature review confirms the broad spectrum of causes of specific back pain in childhood and adolescence. A total of 733 articles dealt with specific causes of back pain, of which 527 individual articles contained sufficient information to be included for qualitative analysis (see Supplementary Material). Table S1 (see Supplementary Material) provides an overview of the relevant causes of disease assigned to different disease groups with possible accompanying symptoms derived from these publications. A summarizing descriptive account of warning signs (red flags) is presented in Table 1 based on the systematic recording in Table S1.

Specific causes of back pain in children and adolescents can occur and be elicited for almost all disease groups at any age. This is especially true for infectious diseases, oncological diseases, and congenital structural diseases of the spine. In adolescence, however, there is a higher risk for the manifestation of non-specific back pain [13,14,15]. Thus, the occurrence of back pain in children in the first decade of life can be formulated as an independent red flag for specific causes of disease.

In almost all disease groups of specific causes, neurological signs may be detectable in the clinical examination (see Table S1). Most common are motor and sensory disturbances of the extremities, radicular pain, and bladder or bowel disturbances. Fever is seen in infectious diseases, neoplasms, rheumatic diseases, and vascular diseases. Local swelling and lymph node enlargement may occur in infectious diseases and neoplasms. In addition, visible structural changes of the spine, palpable step deformity, or hypermobility of the joints are red flags for both congenital and acquired structural diseases of the spine. Evidence of inflammatory signs of disease, such as arthritis, sacroiliitis, enthesitis, or dermal vasculitis are red flags for rheumatic or autoinflammatory disease. Arterial hypertension may be a warning sign of vascular disease as a specific cause of child and adolescent back pain. Concomitant pain locations are detectable in a wide range of underlying conditions.

None of the red flags presented is specific to a particular disease. It is important to note that red flags are optional features for the different causes of the underlying diseases and are not obligatory. Health care professionals need to apply extensive knowledge of specific back pain and consider all relevant factors. A comprehensive evaluation of the medical history and a clinical examination are the ideal basis for further diagnosis and differentiation from non-specific back pain in children and adolescents.

**Recommendation 1: Detection of the red flags presented in Table 1 should prompt diagnosis for specific causes of disease.** (*A, expert consensus 100%)*

**Recommendation 2: If one or more red flags for specific causes of disease are detected in children or adolescents, extended diagnostics should be performed.** (*A, expert consensus 100%)*

#### 3.1.2. Risk Factors for Non-Specific Back Pain in Childhood and Adolescence

The data analysis included three systematic reviews, 16 longitudinal studies, and 57 cross-sectional studies. Table 2 summarizes the risk factors for non-specific back pain in childhood and adolescence derived from these studies. To ensure validity of the risk factors, results of systematic reviews and longitudinal studies were the primary focus. Evidence-based risk factors comprise demographic characteristics, competitive sports, previous pain episodes, and psychosocial factors. Potential risk factors for non-specific back pain in children and adolescents are factors for which most studies demonstrated an association with pain. These potential risk factors include high levels of sports, family history of back pain, workplace factors, and health behavior. Further research is recommended to clarify the significance of these factors. No association has been found between pediatric back pain and physical factors (such as height, weight, BMI, posture; [4,13,14,16,17,18], carrying a school bag or backpack [14,19], ethnicity [13,20,21,22] or socioeconomic status [20,22]).

**Recommendation 3: When diagnosing non-specific back pain in adolescence, the presence of any evidence-based risk factor presented in Table 2 should be assessed.** *(A, level of evidence 1–2, consensus 100%)*

#### 3.1.3. Risk Factors for Chronicity of Non-Specific Back Pain (“Yellow Flags”) in Children and Adolescents

To examine risk factors for the chronicity of non-specific back pain, longitudinal studies are required that consider the course of back pain at several points in time. The identified systematic reviews include both longitudinal and cross-sectional studies. Therefore, the data presented include three longitudinal studies that fulfil the aforementioned criteria [18,25,30]. Table 3 summarizes evidence-based risk factors for chronicity of non-specific back pain in childhood and adolescence derived from these studies. They comprise demographic data, psychosocial factors, and health behavior.

**Recommendation 4: When risk factors for a chronic course are identified in pediatric patients with non-specific back pain (see Table 3), this should be reflected in treatment planning.** *(A, level of evidence 2, consensus 100%)*

#### 3.1.4. Prognosis of Non-Specific Back Pain in Childhood and Adolescence

Two systematic reviews and 22 longitudinal studies were analyzed regarding the prognosis of non-specific back pain. Evidence-based statements on the prognosis of non-specific back pain in childhood and adolescence are currently not possible due to methodological limitations and lack of comparability of studies. Data on recurrent and persistent courses in this age group vary between 10% and 30% [32,33,34,35]. The proportion of more severely impaired children and adolescents is between 5 and 10% of those affected [18,32].

When considering manifest symptoms, there is often no clear distinction between risk factors for recurrent or chronic back pain and the prognosis. An approach used in some studies is cluster analysis that uses a trajectory model to investigate different courses of a disease (e.g., increasing, decreasing, recurrent, or persistent symptoms) and their influencing factors. Table 4 summarizes evidence-based prognostic factors for the persistence of non-specific back pain in childhood and adolescence derived from such studies.

**Recommendation 5: When prognostic factors of a chronic course are identified in pediatric patients with non-specific back pain (see Table 4), this should be reflected in treatment planning.** *(B, level of evidence 1–2, consensus 100%)*

### 3.2. Diagnostics

The data analysis for diagnostics included four systematic reviews and eight individual studies. Anamnesis and physical examination can identify concomitant symptoms and risk factors for the presence of specific back pain in children and adolescents. The broad spectrum of specific causes of back pain in children and adolescents (see Supplementary Table S1) confirms the need for accurate differential diagnostics. Systematic history and physical examination guide further diagnostics.

**Recommendation 6: The initial consultant for back pain in children and adolescents should be conducted by a primary care pediatrician or a general practitioner.** (*A, expert consensus 100%)*

#### 3.2.1. Medical History

Medical history should probe pain characteristics, localization, duration, intensity, and locations. Evidence for inflammatory causes of disease such as fever, inflammatory manifestations of the skin or other organs, or evidence for joint inflammation can also often be evident in the medical history. The medical history should systematically record possible red flags for specific causes of disease (see Table 1), and note any other accompanying symptoms, neurological signs, movement disorders, or muscle weakness.

**Recommendation 7: The medical history should systematically record pain characteristics, onset of symptoms, possible triggers, concomitant symptoms, and red flags for underlying diseases.** (*A, expert consensus 100%)*

**Recommendation 8: The medical history in children and adolescents with back pain should includean assessment of possible non-specific back pain. Evidence-based risk factors for non-specific back pain should be considered (see Table 2).** (*A, expert consensus 100%)*

#### 3.2.2. Physical Examination

Possibilities and limitations of physical examination for diagnosing pediatric back pain are emphasized in several studies [36,37,38,39,40]. The physical examination is used to record the posture, position, and movement function of the entire spine and the rest of the musculoskeletal system. In particular, neurological functions and possible warning signs such as radicular disturbances of motor function or sensitivity, limitations of muscle strength, disturbances of coordination, sensory deficits, and clinical signs of bladder or bowel dysfunction should be considered and recorded. Beyond the local and neurological examinations, the physical examination includes assessment of neighboring structures and organs, skin, and joints.

**Recommendation 9: Physical examination of children and adolescents with back pain should include inspection, testing of locomotor function, and a neurological examination. Red flags for underlying diseases should be systematically considered and assessed (see Table 1).** (*A, expert consensus 100%)*

#### 3.2.3. Diagnostic Imaging

Compared to magnetic resonance imaging (MRI), native X-ray diagnostics have a low sensitivity in the general differential diagnosis of specific causes of back pain in childhood and adolescence [5,41]. Both structural diseases and involvement of the central nervous system and adjacent soft tissue structures and organs can be detected by MRI [5,39,41]. However, depending on the respective differential diagnostic problem, an individual selection of the primary imaging examination or its combination is most useful, ideally with evaluation by radiologists with expertise in pediatric radiology. Depending on the differential diagnosis, MRI examination with contrast may be useful.

**Recommendation 10: If the history and physical examination reveal evidence of a specific cause of disease in pediatric back pain, a targeted imaging examination should be performed.** (*A, level of evidence 2, consensus 100%)*

**Recommendation 11: Depending on the symptoms and findings, an X-ray examination and/or MRI is recommended. The MRI examination protocol should be adapted to age-specific questions and include the paravertebral soft tissues. The MRI should be evaluated by a radiologist with expertise in pediatric radiology.** (*A, expert consensus 100%*)

#### 3.2.4. Laboratory Tests

Systematic studies on the general use of laboratory tests in the differential diagnosis of back pain in childhood and adolescence are not available. If specific diseases are detected during a diagnostic algorithm, laboratory tests are conducted as supplementary diagnostics after the imaging examinations. These confirm or assess the activity and severity of the disease, detect complications, or monitor the initiated therapy. A recommendation for routine use of laboratory diagnostics in the differential diagnosis of specific versus non-specific back pain in childhood and adolescence cannot be formulated based on the current state of the literature.

#### 3.2.5. Screening of Psychological and Social Risk Factors

Screening of psychological and social risk factors is performed as part of a systematic medical history. Standardized questionnaires are suitable to systematically record the impact of pain on everyday family and school life, physical activities, and social and leisure behaviors. Validated questionnaires such as the RCADS (Revised Children’s Anxiety and Depression Scale; [42,43]) are suitable as screening instruments. While questionnaires do not replace clinical psychological diagnostics, they can be helpful as screening instruments. If the medical history and clinical examination confirm suspicions of significant psychosocial risk factors, comprehensive psychological assessment should be carried out [4,13,14,15,17,18,23,24,26]. Screening of non-specific back pain in children and adolescents should also consider the presence of risk factors for a chronic course (see Table 3).

**Recommendation 12: If there is evidence of psychosocial risk factors, psychological diagnostic tests should be performed.** (*A, level of evidence 1–2, consensus 100%)*

**Recommendation 13: In the case of a chronic course of non-specific back pain in pediatric patients, medical and psychological diagnoses should be made in a specialized facility such as an outpatient pain clinic for children and adolescents.** (*A, expert consensus 100%*)

#### 3.2.6. Multidisciplinary Assessment

If specific causes of disease in children and adolescents with back pain are confirmed, specialist co-evaluations may guide further diagnostic and therapeutic measures. If the attribution of a specific cause of disease is uncertain, such as unclear findings in advanced imaging, a multidisciplinary assessment may help plan and implement further exploratory measures. To date, there are no systematic clinical studies on this topic. If psychosocial risk factors are identified in the screening process, comprehensive psychological diagnostics should be carried out as part of a multidisciplinary assessment.

**Recommendation 14: If the diagnosis confirms indications of a specific cause of the disease in children and adolescents with back pain, a specialized multidisciplinary medical evaluation (e.g., pediatric surgery, pediatric hematology and oncology, pediatric orthopedics, pediatric radiology, pediatric rheumatology, neurosurgery, or pediatric neurology) should be performed.** (*B, expert consensus 100%)*

**Figure 2:**
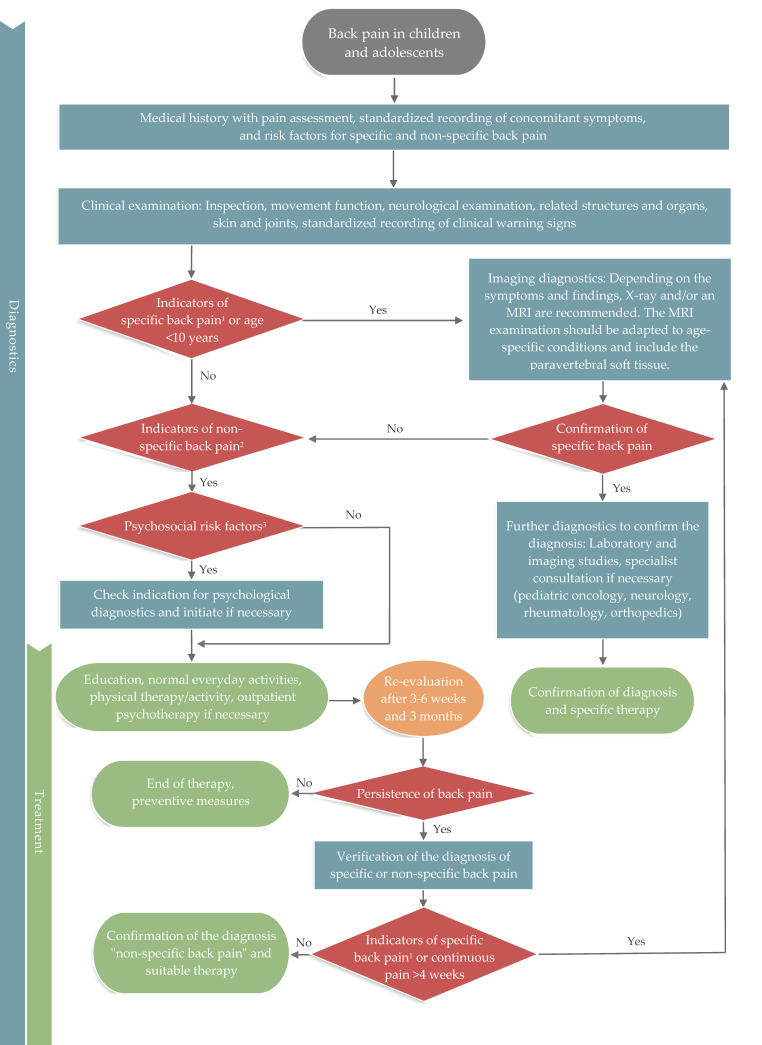
Diagnostic algorithm for back pain in children and adolescents. Green box = treatment; blue box = diagnostic measures; red box = diagnostic decision. ^1^ Red flags for specific back pain (Chapter 3.1.1, Table 1), ^2^ Risk factors for non-specific back pain (Chapter 3.1.2, Table 2), ^3^ Low life satisfaction, anxiety, depression, low self-esteem (see Table 2).

In summary, according to current studies and the above recommendations, the diagnostic algorithm presented in Figure 2 can be used to differentiate specific and non-specific back pain.

## 4. Discussion

This guideline aimed to provide evidence-based, age-specific recommendations for optimizing diagnosis that differentiates specific from non-specific back pain in children and adolescents. In summary, few studies with high methodological quality exist. As a result, only three evidence-based recommendations could be formulated for causes, red flags and risk factors for back pain, while two recommendations are based on expert consensus. Regarding diagnostics, eight expert consensus recommendations and one evidence-based recommendation could be provided. Despite the importance of adequate diagnosis and treatment of back pain in children and adolescents, this lack of scientific evidence confirms the deficit in research investment in this area [4].

The literature search on back pain in childhood and adolescence confirms an extensive number of disorders that need to be considered in the differential diagnosis (see Supplementary Table S1). So far, there are no systematic studies that have tested the sensitivity and validity of red flags for differentiating specific and non-specific back pain in these patients. Systematic reviews of concomitant symptoms and diagnostics of specific causes exist only for certain disease groups, such as central and spinal CNS tumors [36], juvenile adolescent scoliosis [44] and spondylolysis and spondylolisthesis [45]. A diagnostic algorithm developed using confirmed red flags can aid the reliable detection of infrequent specific causes, reducing the risk of overdiagnosis and allowing clinicians to provide timely treatment. Avoiding overdiagnosis is particularly important considering the increasing prevalence of non-specific back pain in adolescence. Due to the lack of research regarding red flags of back pain, this guideline was based on a comprehensive search of described warning signs, including anamnestic information, concomitant symptoms, clinical signs, and pain characteristics. None of the red flags identified in this guideline is specific to a particular disease. It is also remarkable that in almost all disease groups of specific causes, neurological signs—especially motor and sensory disturbances of the extremities, radicular pain, and bladder or bowel disturbances—may be detectable in the clinical examination (see Supplementary Table S1). The present analysis of red flags only partially confirms previous recommendations based on clinical experience. For example, our recommendations correspond with previous publications regarding fever, lymphadenopathy and neurological signs [7,8,9]. For demographic data, medical history, and pain characteristics, however, expert opinions differ from each other and thus also from the present analysis [7,8,9,10,46]. Yet, our recommendations can be an important tool for future studies regarding the evaluation and validation of these identified red flags in the differential diagnosis of specific versus non-specific back pain in children and adolescents.

In contrast to specific back pain, there are numerous publications and high-quality systematic reviews on risk factors for non-specific back pain in childhood and adolescence [4,13,19]. They made an evidence-based description of risk factors feasible. These risk factors concern medical history, demographic data, and psychosocial factors; the latter two can also be risk factors for a chronic course of non-specific back pain [18,25,30]. These factors should be considered during diagnostics and therapy planning. On the other hand, the methodologies of available studies vary considerably regarding the prognosis of non-specific back pain in childhood and adolescence. There is a lack of reliable information on the prognosis of recurrent and persistent back pain in children and adolescents. Important parameters such as pain intensity, degree of impairment, and treatment are missing in most publications. Future studies should focus on identifying factors that enable predicting the course of non-specific back pain into adulthood. This would help the timely identification and treatment of children and adolescents at risk of a prolonged course of back pain that could affect their well-being and work ability in adulthood.

Despite the importance of reliable diagnosis and differentiation of specific and non-specific back pain in children and adolescents, there are few systematic studies on this topic [5,36,39,40,41]. The final decision of an extended, targeted evaluation is primarily based on the medical history and physical examination. These clinical examinations record accompanying symptoms and other warning signs, and may indicate a potential specific cause of the disease. The possible outcomes and limitations of initial clinical diagnostics are described for some underlying diseases, such as spinal CNS tumors [36], juvenile adolescent scoliosis [37,40], and spondylolysis [39]. The systematic analysis of red flags presented in this guideline enabled the creation of a diagnostic algorithm (Figure 2). Although this diagnostic algorithm is based on the best available evidence, the pool of studies is poor and includes many case studies. While outside the scope of the present guideline, future studies should assess the sensitivity and validity of this diagnostic algorithm.

Diagnostic imaging is indispensable for the detection and confirmation of specific causes of back pain in children and adolescents [5,41]. In studies examining the sensitivity of a diagnostic algorithm, MRI is significantly more sensitive than x-ray in detecting specific causes of back pain. On the other hand, radiography has advantages in the diagnosis of structural diseases of the spine [39]. Therefore, the present recommendations on imaging do not specify distinct methods. To avoid overdiagnosing patients with non-specific back pain in adolescence, the present guideline does not recommend routinely using imaging in the diagnostic algorithm. Diagnostic imaging is best used to detect red flags or continuous pain for more than four weeks.

Psychosocial factors are particularly informative for the diagnosis of non-specific back pain in childhood and adolescence, as they are associated with the occurrence and chronicity of back pain [4,18,25]. Therefore, evidence of psychosocial risk factors in non-specific back pain in childhood and adolescence motivates implementing psychological diagnostics. Multidisciplinary assessment has proven helpful in everyday clinical practice for cases where the attribution of a specific cause of disease is unclear or if psychosocial risk factors are evident. However, no evidence-based studies exist in this field.

In summary, studies that validate red flags for specific causes of back pain and that review the diagnostic algorithm are urgently needed to promote reliable diagnosis of back pain in children and adolescents in the future.

## Figures and Tables

**Table 1:** Red flags for specific back pain in children and adolescents.

Category	Red Flag
Demographic data	Age < 10 years
Medical history	Trauma, respiratory arrest after trauma
	Onset of back pain associated with exercise
	Previous or current glucocorticoid therapy
	Pre-existing and concomitant medical conditions
Neurological symptoms	Motor or sensory disturbances of the extremities
	Radicular pain
	Bladder or bowel dysfunction
Other clinical signs	Fever
	Local swelling
	Lymph node enlargement
	Externally apparent structural changes of the spine
	Palpable step deformity
	Joint hypermobility
	Inflammatory signs of disease (arthritis, enthesitis, cutaneous vasculitis)
	Arterial hypertension
Pain characteristics/other pain locations	Compression pain or localized pressure pain
	Pain in the head, thorax, abdomen, flanks, extremities, glutes, or pelvis
	Arthralgia or myalgia

Note: Red flags are optional features for the different causes of the underlying diseases and are not obligatory.

**Table 2:** Evidence-based and potential risk factors for non-specific back pain in children and adolescents.

Category	Risk Factor	References
**Evidence-based risk factors**		
Demographic data	Increasing age in adolescence	[13,14,15]
	Female gender	[4,13,15,17,18,23]
Medical history	Competitive sports	[4,13,14]
	Previous pain episodes	[4,17,24]
Psychosocial factors	Low life satisfaction	[18]
	Anxiety	[15,25]
	Depression	[17,18,23,25,26]
	Low self-esteem	[25]
**Potential risk factors**		
Medical history	High levels of sports activity and technical sports	[14,27,28]
	Family history of back pain	[4,13]
Workplace factors	e.g., sitting, lifting and carrying, posture	[24,29]
Health behavior	Smoking ^1^	[4,13,23,30]
	Short sleep duration, inadequate sleep quality	[31]

^1^ independent of possible confounding factors such as socioeconomic status, physical activity, BMI, and different psychosocial factors [23,30].

**Table 3:** Evidence-based risk factors for chronicity of non-specific back pain in children and adolescents.

Category	Risk Factor	References
Demographic data	Female gender	[25]
Psychosocial factors	Low life satisfaction	[18,25]
	Anxiety	[25]
	Depression	[25]
	Low self-esteem	[25]
Health behavior	Regular smoking ^1^	[30].

^1^ independent of possible confounding factors such as socioeconomic status, physical activity, BMI, and depressive mood [30]

**Table 4:** Evidence-based prognostic factors for the persistence of non-specific back pain in children and adolescents.

Category	Prognostic Factor	References
Demographic data	Female gender	[18,32]
Psychosocial factors	Low life satisfaction	[18]
	Depression	
	Somatization disorder	
Co-occurring diseases	Asthma ^1^	[3]
	Headache	[3,32]

^1^ independent of possible confounding factors age and gender [3].

## Supplementary Materials

The following are available online at https://www.mdpi.com/article/10.3390/children9020192/s1, Table S1: Concomitant anamnestic and symptomatic findings for specific back pain recorded in different disease groups in childhood and adolescence with detailed list of references.
