# Supplementary material for: Etiology, Risk Factors, and Diagnosis of Back Pain in Children and Adolescents: Evidence- and Consensus-Based Interdisciplinary Recommendations
Source: Children (Basel). 2022 Feb 2;9(2):192. doi: 10.3390/children9020192 (PMC8870422; doi:10.3390/children9020192)
Supplement: Supplementary file 1 [file children-09-00192-s001.zip › children-1542622-supplementary.pdf]

Supplementary Table S1. Concomitant anamnestic and symptomatic findings for specific back pain recorded in different disease groups in childhood and adolescence.

Disease group	General symptoms, medical history	Neurological symptoms	Other pain locations	Local findings	Other
Infectious disease	Fever, weakness	Gait or other movement disorder, radicular symptoms, bladder or bowel dysfunction	Headache, abdominal or joint pain, myalgias	Local swelling, lymph node enlargement	Cough, nausea, diarrhea
Neoplasms/ spine tumors	Fever	Gait or movement disorder of the arms, radicular symptoms, bladder or bowel dysfunction	Abdominal, leg, or hip pain	Lymph node enlargement, paravertebral swelling	Pathologic fracture, respiratory insufficiency
Neoplasms / tumors of spinal cord, spinal nerves or paraspinal ganglia	Fever,<br>respiratory insufficiency	Weakness with movement disorder of extremities, radicular symptoms, bladder or bowel dysfunction, signs of meningitis, clonus, muscle spasms, changes of character, hallucinations	Extremity pain, headache, or joint pain		
Congenital structural diseases of the spine	Known syndromic or genetic disease	Radicular symptoms, motor or sensory deficits of the extremities, bladder or bowel dysfunction	Thoracic or abdominal pain, gluteal, hip, leg, or joint pain, sprain pain, localized pressure pain	Dysraphia, disproportional growth, increase in scoliosis, kyphosis or lordosis, short trunk, asymmetry of pelvis or spine, torticollis, other deformities, palpable step deformity	Joint hypermobility, limitation of spinal flexion, load instability
Acquired structural diseases of the spine	Trauma, respiratory arrest after trauma, onset associated with athletic activity, glucocorticoid therapy, neoplasms or consumptive chronic diseases, respiratory disorders	Distal neurological deficits, radicular symptoms, bladder or bowel dysfunction	Leg pain, gluteal and hip pain, thoracic and abdominal pain, joint pain, compression pain	Local structural defects in the lumbosacral region, spinal deformity, short trunk, kyphosis, hyperlordosis, palpable step deformity	Limitation of spinal flexion, pelvic tilt, pain on hyperextension, limited knee extension, load instability, joint hypermobility

Supplementary Table S1 continued

Note: The symptoms are optional features for the different causes of the underlying diseases and are not obligatory.

Disease group	General symptoms, medical history	Neurological symptoms	Other pain locations	Local findings	Other
Neurological and neuromuscular diseases	Known cerebral palsy, spinal muscular atrophy, muscular dystrophy, dysraphia, neurofibromatosis, trauma or association with previous athletic activity, hemoglobinopathy	Motor or sensory disorders of the extremities, bladder or bowel dysfunction, acute paraparesis or tetraplegia, radiculopathy, ipsilateral hemiplegia and contralateral sensory disorder, muscle weakness	Headache, extremity pain, radicular pain	Progressive scoliosis	
Rheumatic and inflammatory diseases	Known chronic inflammatory joint disease (JIA), vasculitis or autoinflammatory disease, juvenile dermatomyositis, uveitis, fever, weight loss	Muscle weakness, gait disturbance	Sprain pain, hip pain, arthralgia, bone pain of extremities, thoracic or abdominal pain, compression pain	Peripheral arthritis, sacroiliitis, enthesitis, dermal vasculitis, spinal motion restriction, scoliotic deformity	Uveitis, photosensitive dermatitis
Hematological and vascular diseases	Known hematological disease (thalassemia, sickle cell disease), endocarditis, renal insufficiency, systemic lupus erythematodes, neurofibromatosis, cutaneous angiomas, glomerulonephritis, oral contraception, fever	Radicular pain, leg weakness to paraparesis, sensory disturbances of legs, speech disorder, bladder dysfunction	Thoracic, abdominal or flank pain, pelvic pain, acute bone pain, headache	Progressive scoliosis	Arterial hypertension, vomiting
Abdominal or thoracic diseases	Known cystic fibrosis, premenstrual development	Urinary retention or urinary incontinence	Thoracic or abdominal pain, flank pain, extremity pain		Arterial hypertension, constipation, vomiting, dyspnea

References: Chapter 3.1.1 Causes and red flags for specific back pain in childhood and adolescence

Infectious diseases

1. Abrahamsson, K., Hansson, S., Jodal, U. & Lincoln, K. (1993). Staphylococcus saprophyticus urinary tract infections in children. *European Journal of Pediatrics*, *152*(1), 69–71. https://doi.org/10.1007/BF02072520
2. Acham-Roschitz, B., Aberle, S. W., Pirker, N., Kaulfersch, W., Boehm, M., Roedl, S., Zenz, W., Ring, E. & MacHe, C. J. (2010). Nephropathia epidemica (puumala virus infection) in Austrian children. *Pediatric Infectious Disease Journal*, *29*(9), 874–876. https://doi.org/10.1097/INF.0b013e3181dfbbe5
3. Ahlm, C., Settergren, B., Gothefors, L. & Juto, P. (1994). Nephropathia epidemica (hemorrhagic fever with renal syndrome) in children: Clinical characteristics. *Pediatric Infectious Disease Journal*, *13*(1), 45–49.
4. Ahmad, M., Ekramullah, Ahmad, I. & Asmat Ali, S. (2012). A rare case of intradural spinal hydatid cyst in a paediatric patient. *Jbr-btr*, *95*(2), 87–88.
5. Al-Rahawan, M. M., Gray, B. M., Mitchell, C. S. & Smith, S. D. (2012). Thoracic vertebral osteomyelitis with paraspinous mass and intraspinal extension: an atypical presentation of cat-scratch disease. *Pediatr Radiol*, *42*(1), 116–119. https://doi.org/10.1007/s00247-011-2087-2
6. Araújo, K. C. G. M., Da Rosa E Silva, C., Barbosa, C. S. & Ferrari, T. C. A. (2006). Clinical-epidemiological profile of children with schistosomal myeloradiculopathy attended at the Instituto Materno-Infantil de Pernambuco. *Memorias do Instituto Oswaldo Cruz*, *101*(SUPPL. 1), 149–156.
7. Balaji, G., Thimmaiah, S. & Menon, J. (2014). Brodie's abscess of the posterior ilium: A rare cause for low back pain in children. *BMJ Case Reports*. Scopus. https://doi.org/10.1136/bcr-2014-204684
8. Behera, G., Poduval, M., Patro, D. K. & Sahoo, S. (2017). Brodie's Abscess of Posterior Ilium with Gluteal Syndrome, an Unusual Cause of Paediatric Low Back Pain: A Case Report. *Malays Orthop J*, *11*(2), 68–71. https://doi.org/10.5704/moj.1707.009
9. Bogdanovic, R., Gligic, A., Nikolic, V., Ognjanović, M., Marković, M. & Sarjanović, L. (1994). Belgrade and Hantaan hantaviruses - the causative agents of severe haemorrhagic fever with renal syndrome in children in Serbia. *Pediatric Nephrology*, *8*(3), 299–303. https://doi.org/10.1007/BF00866341
10. Bolivar, R., Kohl, S. & Pickering, L. K. (1978). Vertebral osteomyelitis in children: report of four cases. *Pediatrics*, *62*(4), 549–553.
11. Bonfiglio, M., Lange, T. A. & Min Kim, Y. (1973). Pyogenic vertebral osteomyelitis. Disk space infections. *Clin.orthop.*, *No. 96*, 234–247.
12. Brook, I. (2001). Two cases of diskitis attributable to anaerobic bacteria in children. *Pediatrics*, *107*(2), E26.
13. Buoncristiani, A. M., McCullen, G., Shin, A. Y., Bathgate, B. & Akbarnia, B. A. (1998). An unusual cause of low back pain. Osteomyelitis of the spinous process. *Spine (Phila Pa 1976)*, *23*(7), 839–841.
14. Callan, A. K., Bauer, J. M. & Martus, J. E. (2016). Deep Spine Infection after Acupuncture in the Setting of Spinal Instrumentation. *Spine Deformity*, *4*(2), 156–161. https://doi.org/10.1016/j.jspd.2015.09.045
15. Celebi, S., Sevinir, B., Saraydaroglu, O., Gurpinar, A. & Hacimustafaoglu, M. (2009). Pulmonary actinomycosis. *Indian Journal of Pediatrics*, *76*(2), 236–238. https://doi.org/10.1007/s12098-008-0233-0
16. Chen, P. Y., Chu, H. Y., Shian, W. J., Shu, S. G. & Chi, C. S. (1994). Varicella-zoster virus infection in children with malignancy. *Chinese Medical Journal (Taipei)*, *54*(6), 417–423.
17. Choma, T., Burke, M., Kim, C. & Kakarlapudi, R. (2008). Epidural abscess as a delayed complication of spinal instrumentation in scoliosis surgery: A case of progressive neurologic dysfunction with complete recovery. *Spine*, *33*(3), E76-E80. https://doi.org/10.1097/BRS.0b013e31816245a6
18. Dagli, C. E., Guler, E., Bakan, V., Atilla, N. & Koksal, N. (2009). Miliary tuberculosis accompanying paravertebral tuberculosis abscess in an adolescent. *J Infect Dev Ctries*, *3*(5), 402–404.
19. Dean, D. (2014). Perirectal abscess masquerading as cauda equina syndrome in an otherwise healthy 12-year-old child. *Case Rep Emerg Med*, *2014*, 817124. https://doi.org/10.1155/2014/817124
20. Demaerel, P., Crevits, I., Casteels-Van Daele, M. & Baert, A. L. (1998). Meningoradiculitis due to borreliosis presenting as low back pain only. *Neuroradiology*, *40*(2), 126–127.
21. Donzelli, A., Samara, E., Spyropoulou, V., Juchler, C. & Ceroni, D. (2016). Pediatric Sacroiliitis: Clinical and Microbiologic Differences Between Infants and Children-Adolescents. *Pediatr Infect Dis J*. Vorab-Onlinepublikation. https://doi.org/10.1097/inf.0000000000001502
22. Dornbos, D., 3rd, Morin, J., Watson, J. R. & Pindrik, J. (2016). Thoracic osteomyelitis and epidural abscess formation due to cat scratch disease: case report. *J Neurosurg Pediatr*, *25*(6), 713–716. https://doi.org/10.3171/2016.7.peds1677
23. Dutta, D., Sen, A., Gupta, D., Kuila, P., Chatterjee, D., Sanyal, S. & Das, S. (2018). Childhood Brucellosis in Eastern India. *Indian Journal of Pediatrics*, *85*(4), 266–271. https://doi.org/10.1007/s12098-017-2513-z

24. Eisen, S., Honywood, L., Shingadia, D. & Novelli, V. (2012). Spinal tuberculosis in children. *Arch Dis Child*, *97*(8), 724–729. https://doi.org/10.1136/archdischild-2011-301571
25. Elevli, M., Çivilibal, M., Duru, N. S., Şengül, H., Çölbay, G. & Erdoğan, Y. (2010). Two children with spinal tuberculosis associated with psoas abscess. *Cocuk Enfeksiyon Dergisi*, *4*(3), 110–113. https://doi.org/10.5152/ced.2010.16
26. Fernandez, M., Carrol, C. L. & Baker, C. J. (2000). Discitis and vertebral osteomyelitis in children: An 18-year review. *Pediatrics*, *105*(6), 1299–1304.
27. Fitzgerald, F., Howard, J., Bailey, F. & Soleimanian, S. (2013). Back pain in a previously healthy teenager. *BMJ Case Rep*, *2013.* https://doi.org/10.1136/bcr-2013-200139
28. Giebaly, D. E., Horriat, S., Sinha, A. & Mangaleshkar, S. (2012). Pyomyositis of the piriformis muscle presenting with sciatica in a teenage rugby player. *BMJ Case Rep*, *2012.* https://doi.org/10.1136/bcr.12.2011.5392
29. Goldsmith, D. P., Smergel, E. M., Chadarevian, J. P. de & Fisher, M. C. (1997). Vague back pain in a teenager. *J Clin Rheumatol*, *3*(3), 140–143.
30. Haghighatkhah, H., Jafroodi, Y., Taheri, M. S., Pourghorban, R. & Dehkordy, A. S. (2015). Multifocal skeletal tuberculosis mimicking langerhans cell histiocytosis in a child: A case report with a long-term follow-up. *Iranian Red Crescent Medical Journal*, *17*(12). https://doi.org/10.5812/ircmj.19942
31. Heenan, S. D. & Britton, J. (1995). Septic arthritis in a lumbar facet joint: a rare cause of an epidural abscess. *Neuroradiology*, *37*(6), 462–464.
32. Hernandez-Trujillo, H. S., Dalberg, T., Feder, H., Jr. & Smith, S. R. (2009). A fever of unknown origin workup in the emergency department reveals an unusual pathogen. *Pediatr Emerg Care*, *25*(10), 684–686. https://doi.org/10.1097/PEC.0b013e3181bec8df
33. Hoffer, F. A., Strand, R. D. & Gebhardt, M. C. (1988). Percutaneous biopsy of pyogenic infection of the spine in children. *J Pediatr Orthop*, *8*(4), 442–444.
34. Holliday Iii, P. O., Davis Jr, C. H. & Des Schaffner, L. (1980). Intervertebral disc space infection in a child presenting as a psoas abscess: Case report. *Neurosurgery*, *7*(4), 395–397.
35. Homans, J., Khoo, L., Chen, T., Commins, D. L., Ahmed, J. & Kovacs, A. (2001). Spinal intramedullary cysticercosis in a five-year-old child: case report and review of the literature. *Pediatr Infect Dis J*, *20*(9), 904–908.
36. Hotz, A., Hena, Z. & Gross, E. (2016). A case of back pain that wakes a child from sleep. *JAMA Pediatrics*, *170*(11), 1101–1102. https://doi.org/10.1001/jamapediatrics.2016.0454
37. Hussain, S. & Rathore, M. H. (2007). Cat scratch disease with epidural extension while on antimicrobial treatment. *Pediatr Neurosurg*, *43*(2), 164–166. https://doi.org/10.1159/000098395
38. Hütten, M. & Lassay, E. (2007). Low back pain in a 131/2 year old patient extending over several months. Diagnosis at second view. *Padiatrische Praxis*, *70*(2), 335–342.
39. Ikem, I. C., Bamgboye, E. A. & Olasinde, A. A. (2001). Spinal tuberculosis: a 15 year review at OAUTHC Ile-Ife. *Niger Postgrad Med J*, *8*(1), 22–25.
40. Jansen, B. R. H., Hart, W. & Schreuder, O. (1993). Discitis in childhood: 12-35-year follow-up of 35 patients. *Acta Orthopaedica*, *64*(1), 33–36. https://doi.org/10.3109/17453679308994523
41. Kalkan, E., Cengiz, Ş. L., Çiçek, O., Erdi, F. & Baysefer, A. (2007). Primary spinal intradural extramedullar hydatid cyst in a child. *Journal of Spinal Cord Medicine*, *30*(3), 297–300.
42. Kameda, G., Vieker, S., Hartmann, J., Niehues, T. & Langler, A. (2012). Diastolic heart murmur, nocturnal back pain, and lumbar rigidity in a 7-year girl: an unusual manifestation of lyme disease in childhood. *Case Rep Pediatr*, *2012*, 976961. https://doi.org/10.1155/2012/976961
43. Kang, H. M., Choi, E. H., Lee, H. J., Yun, K. W., Lee, C. K., Cho, T. J., Cheon, J. E. & Lee, H. (2016). The etiology, clinical presentation and long-term outcome of spondylodiscitis in children. *Pediatric Infectious Disease Journal*, *35*(4), e102-e106. https://doi.org/10.1097/INF.0000000000001043
44. Karadereler, S., Orakdogen, M., Kilic, K. & Ozdogan, C. (2002). Primary spinal extradural hydatid cyst in a child: case report and review of the literature. *Eur Spine J*, *11*(5), 500–503. https://doi.org/10.1007/s00586-002-0411-0
45. Karli, A., Belet, N., Danaci, M., Avcu, G., Paksu, Ş., Köken, Ö. & Şensoy, G. (2014). Iliopsoas abscess in children: Report on five patients with a literature review. *Turkish Journal of Pediatrics*, *56*(1), 69–74.
46. Keihani-Douste, Z., Daneshjou, K. & Ghasemi, M. (2006). A quadriplegic child with multiple brain abscesses: Case report of neurobrucellosis. *Medical Science Monitor*, *12*(12), CS119-CS122.
47. Khan, S., Singh, N., Dow, A. & Ramirez-Zamora, A. (2015). Pediatric Acute Longitudinal Extensive Transverse Myelitis Secondary to Neuroborreliosis. *Case Rep Neurol*, *7*(2), 162–166. https://doi.org/10.1159/000438696
48. King, I. C. C., Lawson, G. & Tourret, L. (2017). Atypical back pain in a child: Subcutaneous lumbar abscess associated with chickenpox. *Annals of Pediatric Surgery*, *13*(1), 62–64. https://doi.org/10.1097/01.XPS.0000489146.09928.13
49. Kumar, R., Srivastava, A. K. & Tiwari, R. K. (2011). Surgical management of Pott's disease of the spine in pediatric patients: A single surgeon's experience of 8 years in a tertiary care center. *J Pediatr Neurosci*, *6*(Suppl 1), S101-8. https://doi.org/10.4103/1817-1745.85726
50. Ladhani, S., Phillips, S. D. & Allgrove, J. (2002). Low back pain at presentation in a newly diagnosed diabetic. *Arch Dis Child*, *87*(6), 543–544.
51. Lahat, E., Pillar, G., Ravid, S., Barzilai, A., Etzioni, A. & Shahar, E. (1998). Rapid recovery from transverse myelopathy in children treated with methylprednisolone. *Pediatric Neurology*, *19*(4), 279–282. https://doi.org/10.1016/S0887-8994(98)00065-4

52. Lantsberg, S., Rachinsky, I., Levy, J. & Shulman, H. (2002). A pediatric patient with acute low-back and pelvis pain. *Seminars in Nuclear Medicine*, *32*(3), 233–235. https://doi.org/10.1053/snuc.2002.124182
53. Liew, K. L., Choong, C. S., Liu, P. N., Tsai, D. H., Chen, L. H. & Yang, W. C. (1998). Pyomyositis in childhood: a case report. *Zhonghua Yi Xue Za Zhi (Taipei)*, *61*(8), 488–491.
54. Lighter, J., Kim, M. & Krasinski, K. (2008). Intramedullary schistosomiasis presenting in an adolescent with prolonged intermittent back pain. *Pediatr Neurol*, *39*(1), 44–47. https://doi.org/10.1016/j.pediatrneurol.2008.03.016
55. Lim, S. W., Lim, H. Y., Kannaiah, T. & Zuki, Z. (2017). Streptococcus Constellatus Spondylodiscitis in a Teenager: A Case Report. *Malays Orthop J*, *11*(3), 50–52. https://doi.org/10.5704/moj.1711.004
56. Limaiem, F., Bellil, S., Bellil, K., Chelly, I., Mekni, A., Khaldi, M., Haouet, S., Zitouna, M. & Kchir, N. (2010). Primary hydatidosis of the central nervous system: A retrospective study of 39 Tunisian cases. *Clinical Neurology and Neurosurgery*, *112*(1), 23–28. https://doi.org/10.1016/j.clineuro.2009.09.001
57. Lipsett, S. C. & Neuman, M. I. (2016). Young Child With Abdominal and Back Pain. *Ann Emerg Med*, *68*(6), 780–792. https://doi.org/10.1016/j.annemergmed.2016.04.012
58. Menelaus, M. B. (1964). DISCITIS. AN INFLAMMATION AFFECTING THE INTERVERTEBRAL DISCS IN CHILDREN. *J Bone Joint Surg Br*, *46*, 16–23.
59. Miller, J. H. & Gates, G. F. (1977). Scintigraphy of Sacroiliac Pyarthrosis in Children. *JAMA: The Journal of the American Medical Association*, *238*(25), 2701–2704. https://doi.org/10.1001/jama.1977.03280260031012
60. Morgan, E. R. & Smalley, L. A. (1983). Varicella in immunocompromised children. Incidence of abdominal pain and organ involvement. *Am J Dis Child*, *137*(9), 883–885.
61. Muñiz, A. E. & Evans, T. (2000). Chronic paronychia, osteomyelitis, and paravertebral abscess in a child with blastomycosis. *Journal of Emergency Medicine*, *19*(3), 245–248. https://doi.org/10.1016/S0736-4679(00)00243-2
62. Myojin, S., Kamiyoshi, N. & Kugo, M. (2018). Pyogenic spondylitis and paravertebral abscess caused by Salmonella Saintpaul in an immunocompetent 13-year-old child: A case report. *BMC Pediatrics*, *18*(1). https://doi.org/10.1186/s12887-018-1010-5
63. Nagashima, H., Morio, Y., Nishi, T., Hagino, H. & Teshima, R. (2002). Spontaneous fusion of isthmic spondylolisthesis after discitis: A case report. *Clin Orthop Relat Res*(403), 104–107.
64. Narayan, V., Mohammed, N., Savardekar, A. R., Patra, D. P. & Nanda, A. (2018). Tuberculous spondylolisthesis: a re-appraisal on clinico-radiologic spectrum and surgical treatment paradigm. *World Neurosurg.* Vorab-Onlinepublikation. https://doi.org/10.1016/j.wneu.2018.02.157
65. Nayil, K., Ramzan, A., Wani, A., Nizami, F. & Makhdoomi, R. (2012). Brucella spinal abscess in a teenager. *Neurosurgery Quarterly*, *22*(4), 253–254. https://doi.org/10.1097/WNQ.0b013e3182592dd6
66. Oberdorfer, P., Kongthavonsakul, K. & Lochungvu, H. P. (2012). A 3-year-old boy with kyphosis, back mass and weakness. *BMJ Case Rep*, *2012*. https://doi.org/10.1136/bcr.02.2012.5918
67. Papaliodis, D. N., Roberts, T. T., Richardson, N. G. & Lawrence, J. B. (2014). Spontaneous septic arthritis of the lumbar facet caused by methicillin-resistant Staphylococcus aureus in an otherwise healthy adolescent. *Am J Orthop (Belle Mead NJ)*, *43*(7), 325–327.
68. Peter, J. C., Kieck, C. F. & Villiers, J. C. de (1992). Acute spinal epidural abscess. *Pediatric Surgery International*, *7*(4), 284–288. https://doi.org/10.1007/BF00183982
69. Reinehr, T., Burk, G. & Andler, W. (1999) [Spondylodiscitis in childhood]. *Klin Padiatr*, *211*(5), 406–409. https://doi.org/10.1055/s-2008-1043821 (Erstveröffentlichung Die Spondylodiszitis im Kindesalter.)
70. Reiss-Zimmermann, M., Hirsch, W., Schuster, V., Wojan, M. & Sorge, I. (2010). Pyogenic osteomyelitis of the vertebral arch in children. *J Pediatr Surg*, *45*(8), 1737–1740. https://doi.org/10.1016/j.jpedsurg.2010.04.010
71. Richards, A. L., Bagus, R., Baso, S. M., Follows, G. A., Tan, R., Graham, R. R., Sandjaja, B., Corwin, A. L. & Punjabi, N. (1997). The first reported outbreak of dengue hemorrhagic fever in Irian Jaya, Indonesia. *American Journal of Tropical Medicine and Hygiene*, *57*(1), 49–55.
72. Rockney, R., Ryan, R. & Knuckey, N. (1989). Spinal epidural abscess. An infectious emergency. Case report and review. *Clin Pediatr (Phila)*, *28*(7), 332–334.
73. Rook, J. L., Duffey, D. & DeRoos, S. (2011). A case of autonomically mediated pain due to spinal epidural abscess in an adolescent female. *Pediatric Emergency Care*, *27*(6), 530–532. https://doi.org/10.1097/PEC.0b013e31821d86d5
74. Rubin, R. C., Jacobs, G. B., Cooper, P. R. & Wille, R. L. (1977). Disc space infections in children. *Childs Brain*, *3*(3), 180–190.
75. Ruddy, J. M., Dodson, T. F. & Duwayri, Y. (2014). Open repair of superior mesenteric artery mycotic aneurysm in an adolescent girl. *Annals of Vascular Surgery*, *28*(4), 1032.e21-1032.e24. https://doi.org/10.1016/j.avsg.2013.08.009
76. Rudolph, H., Prieto Dernbach, R., Walka, M., Rey-Hinterkopf, P., Melichar, V., Muschiol, E., Schweitzer-Krantz, S., Richter, J. W., Weiss, C., Böttcher, S., Diedrich, S., Schroten, H. & Tenenbaum, T. (2017). Comparison of clinical and laboratory characteristics during two major paediatric meningitis outbreaks of echovirus 30 and other non-polio enteroviruses in Germany in 2008 and 2013. *European Journal of Clinical Microbiology and Infectious Diseases*, *36*(9), 1651–1660. https://doi.org/10.1007/s10096-017-2979-7

77. Rurnana, M., Mahadevan, A., Khurshid, M. N., Kovoor, J. M.E., Yasha, T. C., Santosh, V., Indira, B. & Shankar, S. K. (2006). Cestode parasitic infestation: intracranial and spinal hydatid disease--a clinicopathological study of 29 cases from South India. *Clinical Neuropathology*, *25*(2).
78. Sarmah, P., Hanumanthappa, A. R. & Chandrappa, N. R. (2014). Serodiagnosis and clinical profile of dengue virus infection in patients presenting to a tertiary care hospital. *Journal of Pure and Applied Microbiology*, *8*(4), 3209–3212.
79. Sayana, M. K., Chacko, A. J. & Mc Givney, R. C. (2003). Unusual cause of infective discitis in an adolescent. *Postgrad Med J*, *79*(930), 237–238.
80. Sayi, E. N. & Mlay, S. M. (1995). Tuberculosis of the spine in children at Muhimbili Medical Centre, Dar es Salaam. *East Afr Med J*, *72*(1), 46–48.
81. Shah, S. S., Goregaonkar, A. A. & Goregaonkar, A. B. (2017). Extensively Drug-resistant Tuberculosis of the Lumbar Spine in a Six-year-old Child: A Case Report. *J Orthop Case Rep*, *7*(2), 40–43. https://doi.org/10.13107/jocr.2250-0685.742
82. Shukla, S. K., Sharma, V., Singh, K. & Trivedi, A. (2010). Primary lumbosacral intradural hydatid cyst in a child. *J Neurosci Rural Pract*, *1*(2), 109–111. https://doi.org/10.4103/0976-3147.71727
83. Sinatra, P. M. & Alander, D. H. (2015). Lemierre Disease: A Case With Multilevel Epidural Abscess and Aggressive Neurological Weakness: Case Report and Literature Review. *Journal of Pediatric Orthopaedics.* Scopus. https://doi.org/10.1097/BPO.0000000000000652
84. Singh, J., Khare, S., Prasad, A. K., Garg, A., Singh, N. P. & Sharma, R. S. (1994). An outbreak of influenza A (H3N2) in Delhi, 1993. *J Commun Dis*, *26*(1), 14–18.
85. Solano, J., Winningham, G., Al Zubeidi, D. & Myers, A. (2016). A 5-year-old with fever, headache, neck stiffness, and leg pain. *Pediatrics*, *138*(5). https://doi.org/10.1542/peds.2015-3762
86. Spinola, S. M., Bell, R. A. & Henderson, F. W. (1981). Actinomycosis. A cause of pulmonary and mediastinal mass lesions in children. *Am J Dis Child*, *135*(4), 336–339.
87. Strober, J. B., Zuppa, A., Brooks-Kayal, A. R. & Ross, K. (1999). A 15-year-old with back pain, fever, and leg numbness. *Seminars in Pediatric Neurology*, *6*(3), 190–195. https://doi.org/10.1016/S1071-9091(99)80012-6
88. Tannous, R. & Grose, C. (2011). Calculation of the anterograde velocity of varicella-zoster virions in a human sciatic nerve during shingles. *Journal of Infectious Diseases*, *203*(3), 324–326. https://doi.org/10.1093/infdis/jiq068
89. Tassinari, D., Forti, S., Torella, M. & Tani, G. (2013). A special case of lower back pain in a 3-year-old girl. *BMJ Case Reports.* Scopus C7 - 4796. https://doi.org/10.1136/bcr.09.2011.4796
90. Tomaszewski, D. & Avella, D. (1999). Vertebral osteomyelitis in a high school hockey player: A case report. *J Athl Train*, *34*(1), 29–33.
91. Vergori, A., Cerase, A., Migliorini, L., Pluchino, M. G., Oliveri, G., Arrigucci, U., Luca, A. de & Montagnani, F. (2015). Pediatric spinal epidural abscess in an immunocompetent host without risk factors: Case report and review of the literature. *IDCases*, *2*(4), 109–115. https://doi.org/10.1016/j.idcr.2015.09.008
92. Williams, T. M. & Conrad, D. A. (2002). Spinal abscess and chronic lobar atelectasis in a 6-year-old boy. *Infections in Medicine*, *19*(9), 425-427+435.
93. Wolfe, M. W. & Bennett, J. T. (1997). Pyomyositis with toxic shock syndrome presenting as back pain and fever: a case report and literature review. *Am J Orthop (Belle Mead NJ)*, *26*(2), 135–137.
94. Wu, S. Y., Wei, T. A. S., Chen, Y. C. & Huang, S. W. (2012). Vertebral osteomyelitis complicated by iliopsoas muscle abscess in an immunocompetent adolescent: Successful conservative treatment. *Orthopedics*, *35*(10), e1576-e1580. https://doi.org/10.3928/01477447-20120919-34
95. Yazici, N., Yalçin, B., Cila, A., Alnay, A. & Büyükpamukçu, M. (2005). Discitis following lumbar puncture in non-Hodgkin lymphoma. *Pediatric Hematology and Oncology*, *22*(8), 689–694. https://doi.org/10.1080/08880010500278764
96. Yea, C., Bitnun, A., Robinson, J., Mineyko, A., Barton, M., Mah, J. K., Vajsar, J., Richardson, S., Licht, C., Brophy, J., Crone, M., Desai, S., Hukin, J., Jones, K., Muir, K., Pernica, J. M., Pless, R., Pohl, D., Rafay, M. F., . . . Yeh, E. A. (2017). Longitudinal Outcomes in the 2014 Acute Flaccid Paralysis Cluster in Canada. *Journal of Child Neurology*, *32*(3), 301–307. https://doi.org/10.1177/0883073816680770
97. Yigit, O., Erol, M., Gayret, O. B., Ustun, I. & Ulas, S. (2016). Coexistence of a ghon complex, pott's disease, and hip arthritis in a child. *Iranian Red Crescent Medical Journal*, *18*(7). https://doi.org/10.5812/ircmj.29800
98. Yildiz, B., Şen, S., Bal, Z. Ş., Erdǒan, D. D., Korkmaz, M. & Vardar, F. (2013). Epidemiological, laboratory and clinical Features of childhood hydatid disease. *Cocuk Enfeksiyon Dergisi*, *7*(2), 53–56. https://doi.org/10.5152/ced.2013.15
99. Yilmaz, B., Ozdemir, G., Aktas, E., Komur, B., Alfidan, S., Memisoglu, S. & Duymus, T. M. (2016). Brucellosis Suspicion is the Most Important Criterion for Diagnosis Particularly in Endemic Regions. *Open Orthop J*, *10*, 7–11. https://doi.org/10.2174/1874325001610010007
100. Yoo, K. H. & Choi, Y. (1994). Haemorrhagic fever with renal syndrome in Korean children. *Pediatric Nephrology*, *8*(5), 540–544. https://doi.org/10.1007/BF00858120

Neoplasms / spine tumours

101. Abbas, A. A. H., Felimban, S. K., Husain, A. H., Fryer, C. J. H. & Baker, D. L. (2004). Back pain due to osteoporosis in children treated for acute lymphoblastic leukaemia: Clinical - Radiological manifestations and treatment. *Haema*, *7*(1), 92–97.
102. Akeda, K., Kasai, Y., Kawakita, E., Seto, M., Kono, T. & Uchida, A. (2009). Primary Ewing sarcoma of the spine mimicking a psoas abscess secondary to spinal infection. *Spine*, *34*(9), E337-E341. https://doi.org/10.1097/BRS.0b013e3181995ec2
103. Al Maqdassy, E. G. & Bakdash, M. M. (2005). Osteoid osteoma of the spine is an important cause of back pain: Two cases and review. *Qatar Medical Journal*, *14*(2), 48–51.
104. Alos, N., Grant, R. M., Ramsay, T., Halton, J., Cummings, E. A., Miettunen, P. M., Abish, S., Atkinson, S., Barr, R., Cabral, D. A., Cairney, E., Couch, R., Dix, D. B., Fernandez, C. V., Hay, J., Israels, S., Laverdiere, C., Lentle, B., Lewis, V., . . . Ward, L. M. (2012). High incidence of vertebral fractures in children with acute lymphoblastic leukemia 12 months after the initiation of therapy. *J Clin Oncol*, *30*(22), 2760–2767. https://doi.org/10.1200/jco.2011.40.4830
105. Alqahtani, A., Amer, R. & Bakhsh, E. (2017). Primary Occipital Ewing's Sarcoma with Subsequent Spinal Seeding. *Case Rep Pediatr*, *2017*, 1521407. https://doi.org/10.1155/2017/1521407
106. Amacher, A. L. & Eltomey, A. (1985). Spinal osteoblastoma in children and adolescents. *Child's Nervous System*, *1*(1), 29–32. https://doi.org/10.1007/BF00706727
107. Andersson, C., Österlundh, G., Enlund, F., Kindblom, L. G. & Hansson, M. (2014). Primary spinal intradural mesenchymal chondrosarcoma with detection of fusion gene HEY1-NCOA2: A paediatric case report and review of the literature. *Oncology Letters*, *8*(4), 1608–1612. https://doi.org/10.3892/ol.2014.2364
108. Antillon, F., Behm, F. G., Raimondi, S. C., Kaste, S. C., Sandlund, J. T. & Pappo, A. S. (1998). Pediatric primary diffuse large cell lymphoma of bone with t(3;22)(q27;q11). *J Pediatr Hematol Oncol*, *20*(6), 552–555.
109. Ardern-Holmes, S., Esrick, E., Degar, B., Vergilio, J. A. & Ullrich, N. J. (2011). Back pain and spinal cord compression: An uncommon presentation of childhood acute myeloid leukemia. *Journal of Pediatric Neurology*, *9*(1), 109–113. https://doi.org/10.3233/JPN-2010-0443
110. Aston, J. W., Jr. (1990). Pediatric update #16. The orthopaedic presentation of neuroblastoma. *Orthop Rev*, *19*(10), 929–932.
111. Atas, E., Kesik, V., Kismet, E. & Koseoglu, V. (2013). Primary vertebral lymphoma presenting with fracture. *Indian Pediatr*, *50*(5), 512–513.
112. Avadhanam, P. K., Vuyyur, S. & Panigrahi, M. K. (2010). A rare occurrence of osteoblastoma in a child. *J Pediatr Neurosci*, *5*(2), 153–156. https://doi.org/10.4103/1817-1745.76118
113. Aydeniz, A., Erkutlu, I., AltIndağ, Ö., Küçükoğlu, B. & Gürsoy, S. (2010). Severe neck and back pain in adolescence: Remember osteoblastoma. *Rheumatology International*, *30*(9), 1243–1244. https://doi.org/10.1007/s00296-009-1048-7
114. Aysun, S., Topçu, M., Günay, M. & Topaloğlu, H. (1994). Neurologic features as initial presentations of childhood malignancies. *Pediatric Neurology*, *10*(1), 40–43. https://doi.org/10.1016/0887-8994(94)90065-5
115. Azarpira, N., Javadi, F. & Safarian, A. (2015). Giant cell tumor of the thoracic vertebra: A case report. *Neurosurgery Quarterly*, *25*(2), 264–266. https://doi.org/10.1097/WNQ.0000000000000040
116. Balachandran, H., Sneha, L. M., Menon, G. & Scott, J. (2017). Langerhans cell histiocytosis as an unusual cause of back pain in a child: A case report and review of literature. *Journal of Craniovertebral Junction and Spine*, *8*(4), 384–386. https://doi.org/10.4103/jcvjs.JCVJS_105_17
117. Band, M. E., Sheldon, C., Brancato, J., Parikh, N. S. & D'Alessandri-Silva, C. (2016). A 17-year-old with steroid-resistant nephrotic syndrome. *Pediatrics*, *137*(5). https://doi.org/10.1542/peds.2015-3205
118. Beckers, R., Uyttebroeck, A. & Demaerel, P. (2002). Acute lymphoblastic leukaemia presenting with low back pain. *Eur J Paediatr Neurol*, *6*(5), 285–287.
119. Bjerregaard, L. L. & Rosthooj, S. (2002). Vertebral compression and eosinophilia in a child with acute lymphatic leukemia. *J Pediatr Hematol Oncol*, *24*(4), 313–315.
120. Boretz, R. S. & Lonner, B. S. (2002). Atypical presentation of an osteoid osteoma in a child. *American journal of orthopedics (Belle Mead, N.J.)*, *31*(6), 347–348.
121. Bowers, D. C., Griffith, T., Gargan, L., Cochran, C. J., Kleiber, B., Foxwell, A., Farrow-Gillespie, A., Orlino, A. & Germann, J. N. (2012). Back pain among long-term survivors of childhood leukemia. *J Pediatr Hematol Oncol*, *34*(8), 624–629. https://doi.org/10.1097/MPH.0b013e31827080de
122. Brown, C. W., Jarvis, J. G., Letts, M. & Carpenter, B. (2005). Treatment and outcome of vertebral Langerhans cell histiocytosis at the Children's Hospital of Eastern Ontario. *Canadian Journal of Surgery*, *48*(3), 230–236.
123. Caksen, H., Odabas, D., Demirtas, M., Kiymaz, N., Anlar, O., Unal, O. & Ugras, S. (2004). A case of metastatic spinal Ewing's sarcoma misdiagnosed as brucellosis and transverse myelitis. *Neurol Sci*, *24*(6), 414–416. https://doi.org/10.1007/s10072-003-0199-7
124. Chakrapani, S. D., Grim, K., Kaimaktchiev, V. & Anderson, J. C. (2008). Osteoblastoma of the spine with discordant magnetic resonance imaging and computed tomography imaging features in a child. *Spine (Phila Pa 1976)*, *33*(25), E968-70. https://doi.org/10.1097/BRS.0b013e31818a0271
125. Chen, S. H., Huang, T. J., Hsueh, S., Lee, Y. Y. & Hsu, R. W. (2002). Unusual bleeding of aneurysmal bone cyst in the upper thoracic spine. *Chang Gung Med J*, *25*(3), 183–189.
126. Choi, S. W., Shin, S. J., Nam, K. W., Seo, K. B. & Kim, G. M. (2012). Primary Ewing sarcoma of lumbar spine in an 8-year-old boy: A case report. *Journal of Pediatric Orthopaedics Part B*, *21*(4), 322–324. https://doi.org/10.1097/BPB.0b013e328351b907

127. Clark, A. & Stanish, W. D. (1985). An unusual cause of back pain in a young athlete. A case report. *Am J Sports Med*, *13*(1), 51–54.
128. Codd, P. J., Riesenburger, R. I., Klimo Jr, P., Slotkin, J. R. & Smith, E. R. (2006). Vertebra plana due to an aneurysmal bone cyst of the lumbar spine. Case report and review of the literature. *Journal of Neurosurgery*, *105 PEDIATRICS*(SUPPL. 6), 490–495.
129. D'Angelo, P., Conter, V., Di Chiara, G., Rizzari, C., Memeo, A. & Barigozzi, P. (1993). Severe osteoporosis and multiple vertebral collapses in a child during treatment for B-ALL. *Acta Haematol*, *89*(1), 38–42.
130. Das, A., Nobi, F., Banik, G. & Kahhar, M. A. (2016). Vertebral compression fractures as a presenting feature of acutelymphoblastic leukemia. *Journal of Medicine (Bangladesh)*, *17*(2), 120–121. https://doi.org/10.3329/jom.v17i2.30078
131. Dashti, A. S., Abdolkarimi, B., Safaei, A., Dehghanian, A. R. & Bazrafshan, A. (2016). Bilateral irritable hip: A rare presentation of leukemia in children. *Archives of Pediatric Infectious Diseases*, *4*(3). https://doi.org/10.5812/pedinfect.28388
132. De La Serna, Francisco Javier, Martinez, M. A., Valdes, M. D., Hornedo, J., Mestre, M. J. & Morales, J. M. (1988). Rhabdomyosarcoma presenting with diffuse bone marrow involvement, hypercalcemia and renal failure. *Medical and Pediatric Oncology*, *16*(2), 123–127.
133. Denis, F. & Armstrong, G. W. (1984). Scoliogenic osteoblastoma of the posterior end of the rib. A case report. *Spine (Phila Pa 1976)*, *9*(1), 74–76.
134. Dho, Y. S., Kim, H., Wang, K. C., Kim, S. K., Lee, J. Y., Shin, H. Y., Park, K. D., Kang, H. J., Kim, I. H., Park, S. H. & Phi, J. H. (2018). Pediatric Spinal Epidural Lymphoma Presenting with Compressive Myelopathy: A Distinct Pattern of Disease Presentation. *World Neurosurgery*. Scopus. https://doi.org/10.1016/j.wneu.2018.03.059
135. Diniz, R. E., Goldenberg, J., Carvalho, J. C. de, Gomes, C. E., Goldenberg, E. D. & Sementille, A. (1995). Lymphoma of unknown origin located in paravertebral muscles: an unusual cause of low back pain in children. *Sao Paulo Med J*, *113*(4), 953–956.
136. Dogan, S., Leković, G. P., Theodore, N., Horn, E. M., Eschbacher, J. & Rekate, H. L. (2009). Primary thoracolumbar Ewing's sarcoma presenting as isolated epidural mass. *Spine Journal*, *9*(1), e9-e14. https://doi.org/10.1016/j.spinee.2007.11.003
137. Doss, V. T., Weaver, J., Didier, S. & Arthur, A. S. (2014). Serial endovascular embolization as stand-alone treatment of a sacral aneurysmal bone cyst. *J Neurosurg Spine*, *20*(2), 234–238. https://doi.org/10.3171/2013.11.spine13412
138. Duncan, R. A. & Hewson, G. C. (2005). Back pain in children: dig a bit deeper. *Eur J Emerg Med*, *12*(6), 317–319.
139. Eder, K. M., Holl, K. & Pumberger, W. (2016). Osteoblastoma of a thoracic vertebra as a differential diagnosis of back pain. *Monatsschrift fur Kinderheilkunde*, *164*(1), 47–51. https://doi.org/10.1007/s00112-015-3407-7
140. Ellenberg, L., Kellerman, J., Dash, J., Higgins, G. & Zeltzer, L. (1980). Use of hypnosis for multiple symptoms in an adolescent girl with leukemia. *J Adolesc Health Care*, *1*(2), 132–136.
141. Garling, R. J., Singh, R., Harris, C. & Haridas, A. (2018). Intradural lumbosacral malignant extrarenal rhabdoid tumor: a case report. *Child's Nervous System*, *34*(1), 165–167. https://doi.org/10.1007/s00381-017-3571-2
142. Greene, S., Hawkins, D. S., Rutledge, J. C., Tsuchiya, K. D., Douglas, J., Ellenbogen, R. G. & Avellino, A. M. (2006). Pediatric intradural extramedullary synovial sarcoma: case report. *Neurosurgery*, *59*(6), E1339; discussion E1339. https://doi.org/10.1227/01.neu.0000245619.24603.96
143. Hafiz, M. G., Islam, A. & Siddique, R. (2010). Back pain and vertebral compression: an unusual presentation of childhood acute lymphoblastic leukemia. *Mymensingh Med J*, *19*(1), 130–136.
144. Hardasmalani, M. D., Naim, F. A., Kroning, D. & Bithoney, W. G. (2003). Emergency department presentations of a rare tumor - Extraosseous cervical paraspinal Ewing's sarcoma. *Journal of Emergency Medicine*, *24*(3), 271–275. https://doi.org/10.1016/S0736-4679(02)00748-5
145. Hoyoux, C., Forget, P., Piette, C., Dresse, M. F., Florkin, B., Rausin, L. & Thiry, A. (2012). Paravertebral Burkitt's Lymphoma in a Child: An Unusual Presentation. *Case Rep Med*, *2012*, 891714. https://doi.org/10.1155/2012/891714
146. Huang, W. D., Yang, X. H., Wu, Z. P., Huang, Q., Xiao, J. R., Yang, M. S., Zhou, Z. H., Yan, W. J., Song, D. W., Liu, T. L. & Jia, N. Y. (2013). Langerhans cell histiocytosis of spine: A comparative study of clinical, imaging features, and diagnosis in children, adolescents, and adults. *Spine Journal*, *13*(9), 1108–1117. https://doi.org/10.1016/j.spinee.2013.03.013
147. Igrutinovic, Z., Medovic, R., Markovic, S., Kostic, G., Raskovic, Z., Tanaskovic-Nestorovic, J., Radovanovic, M. & Vuletic, B. (2016). Rosai–Dorfman disease of vertebra: Case report and literature review. *Turkish Journal of Pediatrics*, *58*(5), 566–571. https://doi.org/10.24953/turkjped.2016.05.020
148. Kar, A., Das, U., Parija, N. C. & Rout, N. (2014). Cytodiagnosis of metastatic Ewing's sarcoma of orbital mass and its confirmation by demonstration of EWS/friend leukemia integration 1 fusion gene. *Journal of Cytology*, *31*(1), 44–46. https://doi.org/10.4103/0970-9371.130700
149. Kayser, R., Mahlfeld, K., Nebelung, W. & Graßhoff, H. (2000). Vertebral collapse and normal peripheral blood cell count at the onset of acute lymphatic leukemia in childhood. *Journal of Pediatric Orthopaedics Part B*, *9*(1), 55–57.
150. Kebudi, R., Ayan, I., Tokuc, G., Darendeliler, E. & Bilge, N. (1998). Epidural spinal cord compression in children with solid tumors. *International Journal of Pediatric Hematology/Oncology*, *5*(5), 373–377.

151. Kehl, D. K., Alonso, J. E. & Lovell, W. W. (1983). Scoliosis secondary to an osteoid-osteoma of the rib. A case report. *Journal of Bone and Joint Surgery - Series A*, *65*(5), 701–703.
152. Khalatbari, M. R., Jalaeikhoo, H., Hamidi, M. & Moharamzad, Y. (2012). Primary spinal epidural rhabdomyosarcoma: A case report and review of the literature. *Child's Nervous System*, *28*(11), 1977–1980. https://doi.org/10.1007/s00381-012-1822-9
153. Khan, I. S., Thakur, J. D., Chittiboina, P. & Nanda, A. (2012). Large sacral osteoblastoma: a case report and review of multi-disciplinary management strategies. *J La State Med Soc*, *164*(5), 251–255.
154. Kim, H. S., Lee, J. E., Jung, S. S., Chon, J., Yoon, D. H., Park, Y. K. & Cho, E. H. (2013). Spinal cord injury due to the giant cell tumor of the second thoracic vertebra: A case report. *Annals of Rehabilitation Medicine*, *37*(2), 269–273. https://doi.org/10.5535/arm.2013.37.2.269
155. Kobayashi, S., Takahashi, J., Sakashita, K., Fukushima, M. & Kato, H. (2013). Ewing sarcoma of the thoracic epidural space in a young child. *Eur Spine J*, *22 Suppl 3*, S373-9. https://doi.org/10.1007/s00586-012-2481-y
156. Küpeli, S., Kara, F., Akyüz, C. & Büyükpamukçu, M. (2010). Eosinophilia and multifocal vertebral involvement with Hodgkin lymphoma. *Pediatric Blood and Cancer*, *55*(3), 560–561. https://doi.org/10.1002/pbc.22493
157. Latha, M. S., Thirugnanasambandam, R. P., Venkatraman, P. & Scott, J. X. (2017). Back pain: An unusual manifestation of acute lymphoblastic leukemia - A case report and review of literature. *J Family Med Prim Care*, *6*(3), 657–659. https://doi.org/10.4103/2249-4863.222020
158. Lefton, D. R., Torrisi, J. M. & Haller, J. O. (2001). Vertebral osteoid osteoma masquerading as a malignant bone or soft-tissue tumor on MRI. *Pediatr Radiol*, *31*(2), 72–75. https://doi.org/10.1007/s002470000378
159. Leonard, M. & McCormack, D. (2006). Solitary eosinophilic granuloma causing spinal cord compression in a child presenting with abdominal pain. *European Journal of Orthopaedic Surgery and Traumatology*, *16*(4), 348–350. https://doi.org/10.1007/s00590-006-0092-1
160. Lmejjati, M., El Attar, H., Layadi, F., Belaabidia, B. & Ali, S. A. B. (2007). Primary Ewing sarcoma of the vertebral column: Case report and literature review. *Journal of Pediatric Neurology*, *5*(3), 251–254.
161. Louis-Ugbo, J., Reddy, A. S. & Heller, J. G. (1998). Delayed radiographic diagnosis of osteoid osteoma in the lumbar spine. *Neuro-Orthopedics*, *23*(1-2), 1–8.
162. Marin, J. R. (2007). A Teenage Girl with Acute Back Pain. *Clinical Pediatric Emergency Medicine*, *8*(1), 65–68. https://doi.org/10.1016/j.cpem.2007.02.007
163. Marushima, A., Matsumaru, Y., Suzuki, K., Takigawa, T., Kujiraoka, Y., Anno, I. & Matsumura, A. (2009). Selective arterial embolization with n-butyl cyanoacrylate in the treatment of aneursymal bone cyst of the thoracic vertebra: A case report. *Spine (Phila Pa 1976)*, *34*(6), E230-4. https://doi.org/10.1097/BRS.0b013e31818f8f7c
164. Massoud, M., Del Bufalo, F., Caterina Musolino, A. M., Schingo, P. M., Gaspari, S., Pisani, M., Orazi, C., Reale, A. & Raucci, U. (2016). Myeloid Sarcoma Presenting as Low Back Pain in the Pediatric Emergency Department. *J Emerg Med*, *51*(3), 308–314. https://doi.org/10.1016/j.jemermed.2016.01.033
165. Mlczoch, L., Attarbaschi, A., Dworzak, M., Gadner, H. & Mann, G. (2005). Alopecia areata and multifocal bone involvement in a young adult with Hodgkin's disease. *Leuk Lymphoma*, *46*(4), 623–627. https://doi.org/10.1080/10428190500032570
166. Mora, J. & Wollner, N. (1999). Primary epidural non-Hodgkin lymphoma: Spinal cord compression syndrome as the initial form of presentation in childhood non-Hodgkin lymphoma. *Med Pediatr Oncol*, *32*(2), 102–105.
167. Mukhopadhyay, P., Gairola, M., Sharma, M. C., Thulkar, S., Julka, P. K. & Rath, G. K. (2001). Primary spinal epidural extraosseous Ewing's sarcoma: Report of five cases and literature review. *Australasian Radiology*, *45*(3), 372–379.
168. Muller, I., Vlach, O., Cienciala, J. & Chaloupka, R. (1999). Low back pain and osteoid osteoma of the spine in childhood. Case study. *Scripta Medica Facultatis Medicae Universitatis Brunensis Masarykianae*, *72*(4), 131–135.
169. O'Brien, J., Ward, E., Doody, O. & Ryan, M. (2009). A case of back pain associated with neurology in a young man. *Ir J Med Sci*, *178*(3), 373–375. https://doi.org/10.1007/s11845-008-0170-y
170. Oguro, K., Sakai, H., Arai, M. & Igarashi, T. (2013). Eosinophilic granuloma of bone: Two case reports. *Brain and Development*, *35*(4), 372–375. https://doi.org/10.1016/j.braindev.2012.06.007
171. Oliveri, M. B., Mautalen, C. A., Rodriguez Fuchs, C. A. & Romanelli, M. C. (1991). Vertebral compression fractures at the onset of acute lymphoblastic leukemia in a child. *Henry Ford Hosp Med J*, *39*(1), 45–48.
172. Omidi-Kashani, F., Hasankhani, E. G. & Rafeemanesh, E. (2014). Sciatica in a five-year-old boy. *Asian Spine J*, *8*(3), 357–360. https://doi.org/10.4184/asj.2014.8.3.357
173. Ono, T., Sakamoto, A., Jono, O. & Shimizu, A. (2018). Osteoid osteoma can occur at the pars interarticularis of the lumbar spine, leading to misdiagnosis of lumbar spondylolysis. *American Journal of Case Reports*, *19*, 207–213. https://doi.org/10.12659/AJCR.907438
174. Pandya, N. A., Meller, S. T., MacVicar, D., Atra, A. A. & Pinkerton, C. R. (2001). Vertebral compression fractures in acute lymphoblastic leukaemia and remodelling after treatment. *Arch Dis Child*, *85*(6), 492–493.

175. Patil, M., Pratinidhi, S. A., Malik, A., Gulati, R. & Joshi, A. R. (2012). Primary B cell Non-Hodgkin's lymphoma presenting with multiple osteolytic bony lesions in skull. *Journal of Clinical and Diagnostic Research*, *5*(7), 1464–1466.
176. Rogalsky, R. J., Black, G. B. & Reed, M. H. (1986). Orthopaedic manifestations of leukemia in children. *J Bone Joint Surg Am*, *68*(4), 494–501.
177. Rothschild, E. J., Savitz, M. H., Chang, T., Worcester, D. & Peck, H. M. (1984). Primary vertebral tumor in an adolescent girl. *Spine (Phila Pa 1976)*, *9*(7), 695–701.
178. Salim, H., Ariawati, K., Suryawan, W. B. & Arimbawa, M. (2014). Osteoporosis resulting from acute lymphoblastic leukemia in a 7-year-old boy: A case report. *Journal of Medical Case Reports*, *8*(1). https://doi.org/10.1186/1752-1947-8-168
179. Samadian, M., Vahidi, S., Khormaee, F. & Ashraf, H. (2009). Isolated, Primary Spinal Epidural Hodgkin's Disease in a Child. *Pediatric Neurology*, *40*(6), 480–482. https://doi.org/10.1016/j.pediatrneurol.2009.01.006
180. Samuda, G. M., Cheng, M. Y. & Yeung, C. Y. (1987). Back pain and vertebral compression: An uncommon presentation of childhood acute lymphoblastic leukemia. *Journal of Pediatric Orthopaedics*, *7*(2), 175–178.
181. Santangelo, J. R. & Thomson, J. D. (1999). Childhood leukemia presenting with back pain and vertebral compression fractures. *Am J Orthop (Belle Mead NJ)*, *28*(4), 257–260.
182. Sarangi, P. K., Mohanty, J., Parida, S., Swain, B. M. & Kumar, S. (2017). Aneurysmal bone cyst of C2 cervical spine presenting as an asymptomatic posterior neck swelling. *Journal of Clinical and Diagnostic Research*, *11*(12), TD01-TD03. https://doi.org/10.7860/JCDR/2017/32660.10990
183. Schmitz, A., Diedrich, O. & Schmitt, O. (2000) [Sacral osteoid osteoma--a rare cause of back pain in childhood and adolescence]. *Klin Padiatr*, *212*(3), 110–112. https://doi.org/10.1055/s-2000-9662 (Erstveröffentlichung Osteoidosteom im Os sacrum--eine seltene Ursache des Ruckenschmerzes beim Kind und Jugendlichen.)
184. Silveri, A., Gaudiano, J. & Lago, G. (2008). Osteoid osteoma: Nidus radioguided spine surgery. *Coluna/ Columna*, *7*(1), 45–50.
185. Simonati, A., Vio, M., Iannucci, A. M., Bricolo, A. & Rizzuto, N. (1981). Lumbar epidural ewing sarcoma - Light and electron microscopic investigation. *Journal of Neurology*, *225*(1), 67–72. https://doi.org/10.1007/BF00313464
186. Siribumrungwong, K., Tangtrakulwanich, B. & Nitiruangjaras, A. (2013). Unusual presentation of giant cell tumor originating from a facet joint of the thoracic spine in a child: A case report and review of the literature. *J Med Case Rep*, *7*, 178. https://doi.org/10.1186/1752-1947-7-178
187. Slavc, I., Urban, C., Kaulfersch, W. & Mutz, I. (1987). Changes in the vertebrae as an initial symptom of leukemia. *Padiatrie und Padologie*, *22*(1), 59–65.
188. Smith, J. R. & Samdani, A. F. (2008). An unusual cause of low back pain in an adolescent. *Jaapa*, *21*(10), 56–57.
189. Srinivasalu, S. & D'Souza, A. (2009). Sacral Ewing's Sarcoma and Challenges in it's Diagnosis on MRI. *J Radiol Case Rep*, *3*(1), 23–26. https://doi.org/10.3941/jrcr.v3i1.79
190. Szudy, A., Litak, J., Zawitkowska, J. & Kowalczyk, J. (2012). Back pain as a first symptom of hematologic malignancy in a 9-year-old girl. *Pediatria Polska*, *87*(1), 95–98. https://doi.org/10.1016/S0031-3939(12)70600-5
191. Togral, G., Arikan, M., Hasturk, A. E. & Gungor, S. (2014). Painful scoliosis due to superposed giant cell bone tumor and aneurysmal bone cyst in a child. *J Pediatr Orthop B*, *23*(4), 328–332. https://doi.org/10.1097/bpb.0000000000000055
192. van Cleve, L., Muñoz, C. E., Riggs, M. L., Bava, L. & Savedra, M. (2012). Pain Experience in Children With Advanced Cancer. *Journal of Pediatric Oncology Nursing*, *29*(1), 28–36. https://doi.org/10.1177/1043454211432295
193. Vázquez-García, B., Barrios, C., Villas, C., San-Julian, M., Maruenda, J. I., Alfonso, M. & Burgos, J. (2012). Ewing's sarcoma of the spine with initial myeloradicular involvement in children and adolescents. *European Orthopaedics and Traumatology*, *3*(3), 189–194. https://doi.org/10.1007/s12570-012-0122-x
194. Verzosa, m. S., Aur, R. J. A., Simone, J. V., Hustu, H. O. & Pinkel, D. P. (1976). Five years after central nervous system irradiation of children with leukemia. *International Journal of Radiation Oncology, Biology, Physics*, *1*(3-4), 209–215. https://doi.org/10.1016/0360-3016(76)90042-0
195. Virayavanich, W., Sirikulchayanonta, V., Jaovisidha, S., Hongeng, S., Laohacharoensombat, W. & Pornkul, R. (2010). Presacral fibrosarcoma in childhood: a case report. *J Med Assoc Thai*, *93*(2), 252–256.
196. Wei, M. A. & RuiXue, M. A. (2006). Solitary spinal eosinophilic granuloma in children. *Journal of Pediatric Orthopaedics Part B*, *15*(5), 316–319. https://doi.org/10.1016/j.cplett.2006.04.066
197. Wei, S. H., Sheen, J. M., Huang, C. B. & Hsiao, C. C. (2001). Primary spinal epidural non-Hodgkin's lymphoma in a child. *Chang Gung Med J*, *24*(12), 820–825.
198. Wilson, P. E., Oleszek, J. L. & Clayton, G. H. (2007). Pediatric spinal cord tumors and masses. *Journal of Spinal Cord Medicine*, *30*(SUPPL. 1), S15-S20.
199. Wong, M., Chung, C. H. & Ngai, W. K. (2002). Hip pain and childhood malignancy. *Hong Kong Med J*, *8*(6), 461–463.
200. Yamamoto, T., Fujita, I., Kurosaka, M. & Mizuno, K. (2001). Sacral radiculopathy secondary to multicentric osteosarcoma. *Spine (Phila Pa 1976)*, *26*(15), 1729–1732.
201. Yavuz, H. & Çakir, M. (2001). Transverse myelopathy: An initial presentation of acute leukemia. *Pediatric Neurology*, *24*(5), 382–384. https://doi.org/10.1016/S0887-8994(01)00258-2
202. Zenonos, G., Jamil, O., Governale, L. S., Jernigan, S., Hedequist, D. & Proctor, M. R. (2012). Surgical treatment for primary spinal aneurysmal bone cysts: experience from Children's Hospital Boston. *J Neurosurg Pediatr*, *9*(3), 305–315. https://doi.org/10.3171/2011.12.peds11253

Neoplasms / tumours of spinal cord, spinal nerves or paraspinal ganglia

203. Bond, J. V. (1975). Abdominal pain caused by metastatic neuroblastoma. *Clin.Oncol.*, *1*(2), 97–99.
204. Bourke, C. J., Lynch, S., Irving, H. & Borzi, P. A. (2002). Retroperitoneal paraganglioma in a child: Resection and vena caval reconstruction. *Pediatric Surgery International*, *18*(5-6), 505–508. https://doi.org/10.1007/s00383-002-0712-8
205. Chen, F., Chiou, S. S., Lin, S. F., Lieu, A. S., Chen, Y. T. & Huang, C. J. (2017). Recurrent spinal primitive neuroectodermal tumor with brain and bone metastases: A case report. *Medicine (Baltimore)*, *96*(46), e8658. https://doi.org/10.1097/md.0000000000008658
206. Cho, J. C. S., Miller, A. & Kettner, N. W. (2009). Cervical Ependymoma in a Male Adolescent With Neck and Back Pain. *Journal of Manipulative and Physiological Therapeutics*, *32*(8), 695–700. https://doi.org/10.1016/j.jmpt.2009.08.021
207. Ekuma, E. M., Ito, K., Chiba, A., Hara, Y., Kanaya, K., Horiuchi, T., Ohaegbulam, S. & Hongo, K. (2017). A Rare Case of Pediatric Lumbar Spinal Ependymoma Mimicking Meningitis. *World Neurosurgery*, *100*, 710.e1-710.e5. https://doi.org/10.1016/j.wneu.2017.02.016
208. Estey, A. & Lim, R. (2010). Sudden-onset back pain and cauda equina syndrome in an adolescent: A case report. *Pediatric Emergency Care*, *26*(9), 672–675. https://doi.org/10.1097/PEC.0b013e3181f054a9
209. Garber, S. T., Bollo, R. J. & Riva-Cambrin, J. K. (2013). Pediatric spinal pilomyxoid astrocytoma: Case report. *Journal of Neurosurgery: Pediatrics*, *12*(5), 511–516. https://doi.org/10.3171/2013.8.PEDS1397
210. Kabler, H. A., Syska, B. E., Springer, B. L. & Singer, J. I. (2008). Ependymoma as a cause of low back pain in a young healthy athlete. *Pediatr Emerg Care*, *24*(10), 685–687. https://doi.org/10.1097/PEC.0b013e3181887e60
211. Karlowee, V., Kolakshyapati, M., Amatya, V. J., Takayasu, T., Nosaka, R., Sugiyama, K., Kurisu, K. & Yamasaki, F. (2017). Diffuse leptomeningeal glioneuronal tumor (DLGNT) mimicking Whipple's disease: a case report and literature review. *Child's Nervous System*, *33*(8), 1411–1414. https://doi.org/10.1007/s00381-017-3405-2
212. Khalatbari, M. R., Hamidi, M., Moharamzad, Y. & Shobeiri, E. (2016). Primary multifocal myxopapillary ependymoma of the flum terminale. *Journal of Neurosurgical Sciences*, *60*(4), 424–429.
213. Komotar, R. J., Carson, B. S., Rao, C., Chaffee, S., Goldthwaite, P. T. & Tihan, T. (2005). Pilomyxoid astrocytoma of the spinal cord: report of three cases. *Neurosurgery*, *56*(1), 191. https://doi.org/10.1227/01.neu.0000146212.95421.b3
214. Kudo, H., Kokunai, T., Kuwamura, K., Tamaki, N., Sawa, H., Izawa, I., Tatsumi, S., Hamano, S. & Matsumoto, S. (1992). Treatment of early recurrent medulloblastoma in children with cisplatin and etoposide: a preliminary report. *Childs Nerv Syst*, *8*(3), 133–135.
215. Lam, C. H. & Nagib, M. G. (2002). Nonteratomatous tumors in the pediatric sacral region. *Spine (Phila Pa 1976)*, *27*(11), E284-7.
216. Lu, Y. C., Fan, H. C., Gao, H. W., Chen, C. M., Jen, Y. M., Cheng, S. N. & Chen, S. J. (2012). Effective radiotherapy cured cauda equina syndrome caused by remitted intracranial germinoma depositing. *Pediatr Neonatol*, *53*(5), 315–319. https://doi.org/10.1016/j.pedneo.2012.07.007
217. Moon, J. H., Jung, T. Y., Jung, S. & Jang, W. Y. (2012). Leptomeningeal dissemination of a low-grade brainstem glioma without local recurrence. *Journal of Korean Neurosurgical Society*, *51*(2), 109–112. https://doi.org/10.3340/jkns.2012.51.2.109
218. Moshfeghi, D. M., Wilson, M. W., Haik, B. G., Hill, D. A., Rodriguez-Galindo, C. & Pratt, C. B. (2002). Retinoblastoma metastatic to the ovary in a patient with Waardenburg syndrome. *Am J Ophthalmol*, *133*(5), 716–718.
219. Nadkarni, T. D., Rekate, H. L. & Coons, S. W. (1999). Plexiform neurofibroma of the cauda equina: Case report. *Journal of Neurosurgery*, *91*(1 SUPPL.), 112–115.
220. Nagib, M. G. & O'Fallon, M. T. (1997). Myxopapillary ependymoma of the conus medullaris and filum terminale in the pediatric age group. *Pediatr Neurosurg*, *26*(1), 2–7.
221. Neinstein, L. S. (1989). Abdominal and flank pain as presenting symptoms of schwannoma. *Journal of Adolescent Health Care*, *10*(2), 143–145. https://doi.org/10.1016/0197-0070(89)90105-8
222. Nisenson, A. & Patterson, G. H. (1945). Spinal cord tumors in children: A study of three cases of ependymoma. *The Journal of Pediatrics*, *27*(4), 315–323.
223. Oake, C., Borg, M. F., Hanieh, A. & Byard, R. W. (2006). Childhood glioblastoma multiforme of the spinal cord. *Australas Radiol*, *50*(4), 360–363. https://doi.org/10.1111/j.1440-1673.2006.01600.x
224. O'Brien, M., Curtis, C., D'Hemecourt, P. & Proctor, M. (2009). A case of persistent back pain and constipation in a 5-year-old boy. *Physician and Sportsmedicine*, *37*(1), 133–137. https://doi.org/10.3810/PSM.2009.04.1694
225. O'Halloran, P. J., Farrell, M., Caird, J., Capra, M. & O'Brien, D. (2013). Paediatric spinal glioblastoma: Case report and review of therapeutic strategies. *Child's Nervous System*, *29*(3), 367–374. https://doi.org/10.1007/s00381-013-2023-x

226. Packer, R. J., Allen, J., Nielsen, S., Petito, C., Deck, M. & Jereb, B. (1983). Brainstem glioma: Clinical manifestations of meningeal gliomatosis. *Annals of Neurology, 14*(2), 177–182. https://doi.org/10.1002/ana.410140204
227. Park, D. H., Park, Y. K., Oh, J. I., Kwon, T. H., Chung, H. S., Cho, H. D. & Suh, Y. L. (2002). Oncocytic paraganglioma of the cauda equina in a child: Case report and review of the literature. *Pediatric Neurosurgery, 36*(5), 260–265. https://doi.org/10.1159/000058430
228. Patibandla, M. R., Kumar, A., Bhattacharjee, S., Sahu, B. P., Uppin, M. & Challa, S. (2012). Dual gliomas with syringomyelia in a child: Case report and literature review. *Pediatr Neurosurg, 48*(3), 168–173. https://doi.org/10.1159/000346258
229. Roushdi, A., Bassal, M. & Johnston, D. L. (2009). Delayed diagnosis in an adolescent with a malignant testicular tumour. *Paediatr Child Health, 14*(6), 393–394.
230. Shirasawa, H., Ishii, K., Iwanami, A., Mikami, S., Toyama, Y., Matsumoto, M. & Nakamura, M. (2014). Pediatric myxopapillary ependymoma treated with subtotal resection and radiation therapy: A case report and review of the literature. *Spinal Cord, 52*(SUPPL. 2), S18-S20. https://doi.org/10.1038/sc.2014.95
231. Sublett, J. M., Davenport, C., Eisenbrock, H., Dalal, S., Jaffar Kazmi, S. A. & Kershenovich, A. (2016). Pediatric Primary Diffuse Leptomeningeal Primitive Neuroectodermal Tumor: A Case Report and Literature Review. *Pediatr Neurosurg*. Epub ahead of print. https://doi.org/10.1159/000452807
232. Svenson, J. & Stephan Stapczynski, J. (1994). Childhood back pain: Diagnostic evaluation of an unusual case. *American Journal of Emergency Medicine, 12*(3), 334–336. https://doi.org/10.1016/0735-6757(94)90153-8
233. Volejnikova, J., Bajciova, V., Sulovska, L., Geierova, M., Buriankova, E., Jarosova, M., Hajduch, M., Sterba, J. & Mihal, V. (2016). Bone marrow metastasis of malignant melanoma in childhood arising within a congenital melanocytic nevus. *Biomedical Papers, 160*(3), 456–460. https://doi.org/10.5507/bp.2016.018
234. Wiegel, T., Grzyska, U., Schwarz, R. & Escherich, G. (1995). Intraspinal metastasis in a patient with a stage I anaplastic Wilm's tumor. *Strahlentherapie und Onkologie, 171*(5), 296–299.
235. Wilne, S., Collier, J., Kennedy, C., Koller, K., Grundy, R. & Walker, D. (2007). Presentation of childhood CNS tumours: a systematic review and meta-analysis. *Lancet Oncology, 8*(8), 685–695. https://doi.org/10.1016/S1470-2045(07)70207-3
236. Wilson, P. E., Oleszek, J. L. & Clayton, G. H. (2007). Pediatric spinal cord tumors and masses. *Journal of Spinal Cord Medicine, 30*(SUPPL. 1), S15-S20.
237. Wong, M., Chung, C. H. & Ngai, W. K. (2002). Hip pain and childhood malignancy. *Hong Kong Med J, 8*(6), 461–463.
238. Wu, C. T., Tsay, P. K., Jaing, T. H., Chen, S. H., Tseng, C. K. & Jung, S. M. (2016). Oligodendrogliomas in Children: Clinical Experiences with 20 Patients. *Journal of Pediatric Hematology/Oncology, 38*(7), 555–558. https://doi.org/10.1097/MPH.0000000000000610
239. Yone, K., Ijiri, K., Hayashi, K., Yokouchi, M., Takenouchi, T., Manago, K., Nerome, Y., Ijichi, O., Ikarimoto, N. & Komiya, S. (2004). Primary malignant peripheral nerve sheath tumor of the cauda equina in a child case report. *Spinal Cord, 42*(3), 199–203. https://doi.org/10.1038/sj.sc.3101567

Neoplasms / others

240. Aydoğan, A., Çorapçioğlu, F., Levent Elemen, E., Gürbüz, Y., Tugay, M. & Öncel, S. (2009). A case report: Gastric adenocarcinoma in childhood. *Turkish Journal of Pediatrics, 51*(5), 489–492.
241. Bahrami, A., Dalton, J. D., Bangalore, S., Henry, C., Krane, J. F., Navid, F. & Ellison, D. W. (2012). Disseminated carcinoma ex pleomorphic adenoma in an adolescent confirmed by application of PLAG1 immunohistochemistry and FISH for PLAG1 rearrangement. *Head Neck Pathol, 6*(3), 377–383. https://doi.org/10.1007/s12105-012-0330-2
242. Braun, P., Serrano, F. M., Kazmi, K. & Alvarez-Garrijo, J. J. (2006). Large subpial lipoma of the dorsolumbar spinal cord in a pediatric patient. *European Journal of Radiology Extra, 58*(3), 63–67. https://doi.org/10.1016/j.ejrex.2006.03.002
243. Brown, J. M., Berkey, B. D. & Brooks, J. A. (2008). Discovery of a renal medullary carcinoma in an adolescent male with sickle cell trait by Tc-99m methylene disphosponate bone scintigraphy. *Clin Nucl Med, 33*(12), 896–900. https://doi.org/10.1097/RLU.0b013e31818bf31d
244. Chaudhary, S. & Sah, J. P. (2017). Hypercalcemia due to nasopharyngeal carcinoma. *Journal of the Nepal Medical Association, 56*(205), 182–185.
245. Choi, S. H., Jeon, H. W., Oh, W. J. & Park, J. K. (2014). Bronchioloalveolar carcinoma in a juvenile rhadomyosarcoma patient. *Korean Journal of Thoracic and Cardiovascular Surgery, 47*(1), 51–54. https://doi.org/10.5090/kjtcs.2014.47.1.51
246. Ded, K. S., Khurana, M. S., Narang, G. S., Gupta, A. K. & Kaur, L. (2012). GIST- A rare tumor in paediatric age group. *Online Journal of Health and Allied Sciences, 11*(1).
247. Diesen, D. L., Price, T. M. & Skinner, M. A. (2008). Uterine leiomyoma in a 14-year-old girl. *Eur J Pediatr Surg, 18*(1), 53–55. https://doi.org/10.1055/s-2007-989299
248. Fang, Y., Lu, J., Lin, J., Zhou, G., Li, Y., Chen, Z., Wei, J., Luo, J. & Chen, W. (2016). Impaired growth and development after sunitinib treatment in a child with locally progressive kidney cancer. *International Journal of Clinical and Experimental Medicine, 9*(2), 4943–4948.

249. Gelabert-Gonzalez, M., Agulleiro-Diaz, J. & Reyes-Santias, R. M. (2002). Spinal extradural angiolipoma, with a literature review. *Childs Nerv Syst, 18*(12), 725–728. https://doi.org/10.1007/s00381-002-0653-5
250. Gun, F., Erginel, B., Ünüvar, A., Kebudi, R., Salman, T. & Celik, A. (2012). Mediastinal masses in children: experience with 120 cases. *Pediatr Hematol Oncol, 29*(2), 141–147. https://doi.org/10.3109/08880018.2011.646385
251. Jha, B. & Choudhary, A. K. (2008). Unusual cause of back pain in an adolescent patient: A case report and natural history of aggressive vertebral hemangioma in children. *Pain Physician, 11*(5), 687–692.
252. Kano, K., Kuwashima, S., Kyo, K., Ito, S., Ando, T. & Ichimura, T. (1996). Steroid-induced epidural lipomatosis in nephrotic children: Early recognition with MR imaging. *Dokkyo Journal of Medical Sciences, 23*(4), 185–191.
253. Keenen, T. L., Buehler, K. C. & Campbell, J. R. (1995). Solitary lymphangioma of the spine. *Spine (Phila Pa 1976), 20*(1), 102–105.
254. Khalatbari, M. R., Hamidi, M. & Moharamzad, Y. (2013). Acute presentation of solitary spinal epidural cavernous angioma in a child. *J Coll Physicians Surg Pak, 23*(5), 364–366.
255. Küpeli, S., Araç, A., Yalçin, B., Sökmensüer, C. & Büyükpamukçu, M. (2008). Lymphangiomatosis in a child: Eight years' follow-up without treatment. *Pediatric Hematology and Oncology, 25*(6), 614–619. https://doi.org/10.1080/08880010802234879
256. Maggiore, U. L. R., Ferrero, S., Bogliolo, S., Fulcheri, E., Musizzano, Y. & Menada, M. V. (2013). A case of large uterine myoma in a 14-year-old girl. *Journal of Gynecologic Surgery, 29*(2), 83–87. https://doi.org/10.1089/gyn.2012.0097
257. Möller, J., Girschick, H. J., Hahn, G. & Pessler, F. (2010). Steroid-induced spinal epidural lipomatosis in pediatric patients. *Zeitschrift für Rheumatologie, 69*(5), 447–449. https://doi.org/10.1007/s00393-010-0608-2
258. Möller, J. C., Cron, R. Q., Young, D. W., Girschick, H. J., Levy, D. M., Sherry, D. D., Kukita, A., Saijo, K. & Pessler, F. (2011). Corticosteroid-induced spinal epidural lipomatosis in the pediatric age group: report of a new case and updated analysis of the literature. *Pediatr Rheumatol Online J, 9*(1), 5. https://doi.org/10.1186/1546-0096-9-5
259. Pretell-Mazzini, J., Chikwava, K. R. & Dormans, J. P. (2012). Low back pain in a child associated with acute onset cauda equina syndrome: A rare presentation of an aggressive vertebral hemangioma: A case report. *Journal of Pediatric Orthopaedics, 32*(3), 271–276. https://doi.org/10.1097/BPO.0b013e318247195a
260. Rajah, G., To, C. Y., Sood, S., Ham, S., Altinok, D., Poulik, J. & Haridas, A. (2014). Epidural spinal cord compression in a patient with blue rubber bleb nevus syndrome. *J Neurosurg Pediatr, 14*(5), 486–489. https://doi.org/10.3171/2014.8.peds13627
261. Rocourt, D. V., Shiels, W. E., Hammond, S. & Besner, G. E. (2006). Contemporary management of benign hepatic adenoma using percutaneous radiofrequency ablation. *J Pediatr Surg, 41*(6), 1149–1152. https://doi.org/10.1016/j.jpedsurg.2006.01.064
262. Sekine, I., Izumi, N. & Hirao, J. (1978). Venous spinal angiomas in childhood. A case report. *Dokkyo Journal of Medical Sciences, 5*(2), 336–344.
263. Singh, P. K., Chandra, P. S., Vaghani, G., Savarkar, D. P., Garg, K., Kumar, R., Kale, S. S. & Sharma, B. S. (2016). Management of pediatric single-level vertebral hemangiomas presenting with myelopathy by three-pronged approach (ethanol embolization, laminectomy, and instrumentation): a single-institute experience. *Child's Nervous System, 32*(2), 307–314. https://doi.org/10.1007/s00381-015-2941-x
264. Skarupa, D. J., Ellison, E. C., Vitellas, K. M. & Frankel, W. L. (2004). Hepatocellular Adenomatosis is a Rare Entity that may Mimic Other Hepatocellular Lesions. *Annals of Diagnostic Pathology, 8*(1), 43–49. https://doi.org/10.1016/j.anndiagpath.2003.11.010
265. Uzunaslan, D., Saygin, C., Gungor, S., Hasiloglu, Z., Ozdemir, N. & Celkan, T. (2013). Novel use of propranolol for management of pain in children with vertebral hemangioma: Report of two cases. *Child's Nervous System, 29*(5), 855–860. https://doi.org/10.1007/s00381-012-2012-5
266. Vallabha, T., Ishwarappagol, V., Narasanagi, B., Sindgikar, V., Patil, V. & Potekar, R. M. (2017). Prepubertal bilateral giant fibroadenoma of breast with ulceration: A case report. *Journal of Krishna Institute of Medical Sciences University, 6*(4), 109–110.
267. Yilmaz, C. & Aydemir, F. (2018). Thoracic Intramedullary Lipoma in a 3-year-old Child: Spontaneous Decrease in the Size Following Incomplete Resection. *Asian J Neurosurg, 13*(1), 188–190. https://doi.org/10.4103/1793-5482.180965

Congenital and aquired structural diseases of the spine

268. Adib, N., Davies, K., Grahame, R., Woo, P. & Murray, K. J. (2005). Joint hypermobility syndrome in childhood. A not so benign multisystem disorder? *Rheumatology, 44*(6), 744–750. https://doi.org/10.1093/rheumatology/keh557
269. Ahemad, A., Dasgupta, B. & Jagiasi, J. (2008). Intervertebral disc calcification in a child. *Indian Journal of Orthopaedics, 42*(4), 480–481. https://doi.org/10.4103/0019-5413.43401

270. Al Kaissi, A., Ganger, R., Klaushofer, K., Rumpler, M. & Grill, F. (2008). Achondroplasia manifesting as enchondromatosis and ossification of the spinal ligaments: A case report. *Journal of Medical Case Reports, 2*. https://doi.org/10.1186/1752-1947-2-263
271. Assad, A. P., Abreu, A. S., Seguro, L. P., Guedes, L. K., Lima, F. R. & Pinto, A. L. (2014). Spondyloptosis in athlete. *Rev Bras Reumatol, 54*(3), 234–236.
272. Bac, A., Stagraczyński, Ł., Ciszek, E., Górkiewicz, M. & Szczygieł, A. (2009). Efficacy of Kinesiology Taping in the rehabilitation of children with low-angle scoliosis. *Fizjoterapia Polska, 9*(3), 202–210.
273. Basile Júnior, R., Barros Filho, T. E. de, Bonetti, C. L. & Rosemberg, L. A. (1992). Herniation of the lumbar disk in adolescents. *Revista Paulista de Medicina, 110*(2), 51–55.
274. Basu, P. S., Hilali Noordeen, M. H. & Elsebaie, H. (2001). Spondylolisthesis in osteogenesis imperfecta due to pedicle elongation: report of two cases. *Spine (Phila Pa 1976), 26*(21), E506-9.
275. Benli, Í. T., Üzümcügil, O., Aydin, E., Ateş, B., Gürses, L. & Hekimoğlu, B. (2006). Magnetic resonance imaging abnormalities of neural axis in Lenke type 1 idiopathic scoliosis. *Spine, 31*(16), 1828–1833. https://doi.org/10.1097/01.brs.0000227256.15525.9b
276. Bettany-Saltikov, J., Weiss, H. R., Chockalingam, N., Kandasamy, G. & Arnell, T. (2016). A Comparison of Patient-Reported Outcome Measures Following Different Treatment Approaches for Adolescents with Severe Idiopathic Scoliosis: A Systematic Review. *Asian Spine J, 10*(6), 1170–1194. https://doi.org/10.4184/asj.2016.10.6.1170
277. Beutler, W. J., Fredrickson, B. E., Murtland, A., Sweeney, C. A., Grant, W. D. & Baker, D. (2003). The natural history of spondylolysis and spondylolisthesis: 45-Year follow-up evaluation. *Spine, 28*(10), 1027–1035. https://doi.org/10.1097/00007632-200305150-00014
278. Blatter, S. C., Min, K., Huber, H. & Ramseier, L. E. (2012). Spontaneous reduction of spondylolisthesis during growth: a case report. *J Pediatr Orthop B, 21*(2), 160–163. https://doi.org/10.1097/BPB.0b013e328346727b
279. Bradbury, N., Wilson, L. F. & Mulholland, R. C. (1996). Adolescent disc protrusions: A long-term follow-up of surgery compared to chymopapain. *Spine, 21*(3), 372–377. https://doi.org/10.1097/00007632-199602010-00024
280. Buttermann, G. R. & Mullin, W. J. (2008). Pain and disability correlated with disc degeneration via magnetic resonance imaging in scoliosis patients. *Eur Spine J, 17*(2), 240–249. https://doi.org/10.1007/s00586-007-0530-8
281. Carbó, E., Riquelme, Ó., García, A. & González, J. L. (2015). Vertebroplasty in a 10-year-old boy with Gorham–Stout syndrome. *Eur Spine J, 24*, 590–593. https://doi.org/10.1007/s00586-015-3764-x
282. Campbell, M., Dimar, J. R. 2., Glassman, S. D., Puno, R. M. & Johnson, J. R. (1995). Idiopathic juvenile osteoporosis. An unusual cause of back pain in an adolescent. *American journal of orthopedics (Belle Mead, N.J.), 24*(11), 865–869.
283. Çelik, S., Göksu, K., Çelik, S. E. & Emir, C. B. (2011). Benign neurological recovery with low recurrence and low peridural fibrosis rate in pediatric disc herniations after lumbar microdiscectomy. *Pediatric Neurosurgery, 47*(6), 417–422. https://doi.org/10.1159/000338982
284. Choi, B. S., Hong, S. J., Chu, M. A., Lee, S. J., Lee, J. M., Bae, H. I. & Choe, B. H. (2014). Gastrointestinal tract involvement of Gorham's disease with expression of D2-40 in duodenum. *Pediatric Gastroenterology, Hepatology and Nutrition, 17*(1), 52–56. https://doi.org/10.5223/pghn.2014.17.1.52
285. Chromy, C. A., Carey, M. T., Balgaard, K. G. & Iaizzo, P. A. (2006). The Potential Use of Axial Spinal Unloading in the Treatment of Adolescent Idiopathic Scoliosis: A Case Series. *Archives of Physical Medicine and Rehabilitation, 87*(11), 1447–1453. https://doi.org/10.1016/j.apmr.2006.08.325
286. Clarke, N. M. & Cleak, D. K. (1983). Intervertebral lumbar disc prolapse in children and adolescents. *J Pediatr Orthop, 3*(2), 202–206.
287. Dang, L., Chen, Z., Liu, X., Guo, Z., Qi, Q., Li, W., Zeng, Y., Jiang, L., Wei, F., Sun, C. & Liu, Z. (2015). Lumbar Disk Herniation in Children and Adolescents: The Significance of Configurations of the Lumbar Spine. *Neurosurgery, 77*(6), 954–959. https://doi.org/10.1227/NEU.0000000000000983
288. De Lima, M. V., Duarte Júnior, A., Jorge, P. B., Bryk, F. F., Meves, R. & Avanzi, O. (2014). Frequency of spondylolysis and chronic low back pain in young soccer players. *Coluna/Columna, 13*(2), 120–123. https://doi.org/10.1590/S1808-18512014130200405
289. Diab, M., Sharkey, M., Emans, J., Lenke, L., Oswald, T. & Sucato, D. (2010). Preoperative bracing affects postoperative outcome of posterior spine fusion with instrumentation for adolescent idiopathic scoliosis. *Spine, 35*(20), 1876–1879. https://doi.org/10.1097/BRS.0b013e3181ef5c36
290. Dimar, J. R. 2., Campbell, M., Glassman, S. D., Puno, R. M. & Johnson, J. R. (1995). Idiopathic juvenile osteoporosis. An unusual cause of back pain in an adolescent. *American journal of orthopedics (Belle Mead, N.J.), 24*(11), 865–869
291. Donaldson, L. D. (2014). Spondylolysis in elite junior-level ice hockey players. *Sports Health, 6*(4), 356–359. https://doi.org/10.1177/1941738113519958
292. Dua, S. G. & Ali, A. (2016). Bone scintigraphy and CT findings in transverse process apophysitis. *Clinical Nuclear Medicine, 41*(7), 574–575. https://doi.org/10.1097/RLU.0000000000001182
293. Ebersold, M. J., Quast, L. M. & Bianco Jr, A. J. (1987). Results of lumbar discectomy in the pediatric patient. *Journal of Neurosurgery, 67*(5), 643–647.
294. El Rassi, G., Takemitsu, M., Glutting, J. & Shah, S. A. (2013). Effect of sports modification on clinical outcome in children and adolescent athletes with symptomatic lumbar spondylolysis. *Am J Phys Med Rehabil, 92*(12), 1070–1074. https://doi.org/10.1097/PHM.0b013e318296da7e

295. El Rassi, G., Takemitsu, M., Woratanarat, P. & Shah, S. A. (2005). Lumbar spondylolysis in pediatric and adolescent soccer players. *Am J Sports Med, 33*(11), 1688–1693. https://doi.org/10.1177/0363546505275645
296. Elgafy, H., Hart, R. C. & Tanios, M. (2015). Nonconsecutive Pars Interarticularis Defects. *Am J Orthop (Belle Mead NJ), 44*(12), E526-9
297. Falcini, F., Trapani, S., Ermini, M. & Brandi, M. L. (1996). Intravenous administration of alendronate counteracts the in vivo effects of glucocorticoids on bone remodeling. *Calcif Tissue Int, 58*(3), 166–169.
298. Fisher, R. G. & Saunders, R. L. (1981). Lumbar disc protrusion in children. *Journal of Neurosurgery, 54*(4), 480–483.
299. Fortin, C., Grunstein, E., Labelle, H., Parent, S. & Ehrmann Feldman, D. (2016). Trunk imbalance in adolescent idiopathic scoliosis. *Spine J, 16*(6), 687–693. https://doi.org/10.1016/j.spinee.2016.02.033
300. Gelabert-González, M., Prieto-González, A., María Santin-Amo, J., Serramito-García, R. & García-Allut, A. (2009). Lumbar synovial cyst in an adolescent: Case report. *Child's Nervous System, 25*(6), 719–721. https://doi.org/10.1007/s00381-009-0833-7
301. Gelfand, M. J., Strife, J. L. & Kereiakes, J. G. (1981). Radionuclide bone imaging in spondylolysis of the lumbar spine in children. *Radiology, 140*(1), 191–195. https://doi.org/10.1148/radiology.140.1.6454161
302. Gemmel, F., Coningh, A. de, Collins, J. & Rijk, P. (2011). SPECT/CT of osteitis condensans ilii: one-stop shop imaging. *Clin Nucl Med, 36*(1), 59–61. https://doi.org/10.1097/RLU.0b013e3181feefe8
303. Gorsha, O. V., Aplevich, V. M. & Zukow, W. (2017). Efficiency of kinesiotaping application in the complex rehabilitation of children with idiopathic scoliosis. *Journal of Physical Education and Sport, 17*(3), 1154–1157. https://doi.org/10.7752/jpes.2017.03177
304. Greiner, A. K. (2002). Adolescent idiopathic scoliosis: radiologic decision-making. *American Family Physician, 65*(9), 1817
305. Grier, D., Wardell, S., Sarwark, J. & Poznanski, A. K. (1993). Fatigue fractures of the sacrum in children: two case reports and a review of the literature. *Skeletal Radiol, 22*(7), 515–518.
306. Grødahl, L. H. J., Fawcett, L., Nazareth, M., Smith, R., Spencer, S., Heneghan, N. & Rushton, A. (2016). Diagnostic utility of patient history and physical examination data to detect spondylolysis and spondylolisthesis in athletes with low back pain: A systematic review. *Manual Therapy, 24*, 7–17. https://doi.org/10.1016/j.math.2016.03.011
307. Grossman, D. C., Curry, S. J., Owens, D. K., Barry, M. J., Davidson, K. W., Doubeni, C. A., Epling, J. W., Kemper, A. R., Krist, A. H., Kurth, A. E., Landefeld, C. S., Mangione, C. M., Phipps, M. G., Silverstein, M., Simon, M. A. & Tseng, C. W. (2018). Screening for adolescent Idiopathic Scoliosis US preventive services task force recommendation statement. *JAMA - Journal of the American Medical Association, 319*(2), 165–172. https://doi.org/10.1001/jama.2017.19342
308. Haapanen, A., Latvala, A. & Ala-Ketola, L. (1985). Anterior intervertebral disc herniation in young athletes. *Scandinavian Journal of Sports Sciences, 7*(2), 41–44.
309. Haasbeek, J. F. & Green, N. E. (1994). Adolescent stress fractures of the sacrum: Two case Reports. *Journal of Pediatric Orthopaedics, 14*(3), 336–338.
310. Halperin, N., Copeliovitch, L. & Schachner, E. (1983). Radiating leg pain and positive straight leg raising in spondylolysis in children. *J Pediatr Orthop, 3*(4), 486–490
311. Helenius, I., Remes, V., Yrjönen, T., Ylikoski, M., Schlenzka, D., Helenius, M. & Poussa, M. (2005). Does gender affect outcome of surgery in adolescent idiopathic scoliosis? *Spine, 30*(4), 462–467. https://doi.org/10.1097/01.brs.0000153347.11559.de
312. Hession, E. F. & Donald, G. D. (1993). Treatment of multiple lumbar disk herniations in an adolescent athlete utilizing flexion distraction and rotational manipulation. *J Manipulative Physiol Ther, 16*(3), 185–192.
313. Hoashi, J. S., Thomas, S. M., Goodwin, R. C., Gurd, D. P., Hanna, R. & Kuivila, T. E. (2016). Balloon Kyphoplasty for Managing Intractable Pain in Pediatric Pathologic Vertebral Fractures. *J Pediatr Orthop*. Epub ahead of print. https://doi.org/10.1097/bpo.0000000000000886
314. Holcomb, R. R., Worthington, W. B., McCullough, B. A. & McLean, M. J. (2000). Static magnetic field therapy for pain in the abdomen and genitals. *Pediatric Neurology, 23*(3), 261–264. https://doi.org/10.1016/S0887-8994(00)00180-6
315. Hoo, J. J. & Oliphant, M. (2003). Two sibs with brachyolmia type Hobaek: Five year follow-up through puberty. *American Journal of Medical Genetics, 116*(1), 80–84
316. Hopkins, J., Sakai, T., Sairyo, K., Mefford, J., Bhatia, N. N., Tonogai, I., Dezawa, A. & Yasui, N. (2013). Endoscope-assisted excision of a juxtafacet cyst in an adolescent athlete: A case report. *Journal of Neurological Surgery, Part A: Central European Neurosurgery, 74*(SUPPL.1), e66-e69. https://doi.org/10.1055/s-0032-1325635
317. Ishihara, H., Matsui, H., Hirano, N. & Tsuji, H. (1997). Lumbar intervertebral disc herniation in children less than 16 years of age: Long-term follow-up study of surgically managed cases. *Spine, 22*(17), 2044–2049. https://doi.org/10.1097/00007632-199709010-00022
318. Ishikawa, S., Kumar, S. J. & Torres, B. C. (1994). Surgical treatment of dysplastic spondylolisthesis: Results after in situ fusion. *Spine, 19*(15), 1691–1696.
319. Jalanko, T., Helenius, I., Remes, V., Lamberg, T., Tervahartiala, P., Yrjönen, T., Poussa, M. & Schlenzka, D. (2011). Operative treatment of isthmic spondylolisthesis in children: A long-term, retrospective comparative study with matched cohorts. *European Spine Journal, 20*(5), 766–775. https://doi.org/10.1007/s00586-010-1591-7
320. Johnson, D. L., Falci, S. & McLone, D. G. (1990). The diagnosis and treatment of pediatric lumbar spine injuries caused by rear seat lap belts. *Neurosurgery, 26*(3), 434–441.

321. Joncas, J., Labelle, H., Poitras, B., Duhaime, M., Rivard, C. H., Grimard, G. & Leblanc, R. (1997). Back pain in patients with adolescent idiopathic scoliosis(AIS). *Studies in Health Technology and Informatics, 37*, 381–384. https://doi.org/10.3233/978-1-60750-881-6-381
322. Kalevski, S. K., Haritonov, D. G. & Peev, N. A. (2014). Lumbar intraforaminal synovial cyst in young adulthood: case report and review of the literature. *Global Spine J, 4*(3), 191–196. https://doi.org/10.1055/s-0034-1370694
323. Kaloostian, P. E., Kim, J. E., Calabresi, P. A., Bydon, A. & Witham, T. (2013). Clay-shoveler's fracture during indoor rock climbing. *Orthopedics, 36*(3), e381-3. https://doi.org/10.3928/01477447-20130222-31
324. Karlsson, M. K., Moller, A., Hasserius, R., Besjakov, J., Karlsson, C. & Ohlin, A. (2003). A modeling capacity of vertebral fractures exists during growth: an up-to-47-year follow-up. *Spine (Phila Pa 1976), 28*(18), 2087–2092. https://doi.org/10.1097/01.brs.0000084680.76654.b1
325. Kemmochi, M., Sasaki, S. & Ichimura, S. (2018). Association between reduced trunk flexibility in children and lumbar stress fractures. *Journal of Orthopaedics, 15*(1), 122–127. https://doi.org/10.1016/j.jor.2018.01.014
326. Kim, J. H., Kim, S. W. & Kim, H. S. (2012). Congenital osseus bridging of lumbar transverse processes. *J Korean Neurosurg Soc, 52*(2), 159–160. https://doi.org/10.3340/jkns.2012.52.2.159
327. Kim, P., Kim, S. W., Ju, C. I. & Kim, H. S. (2015). Lumbar Disc Herniation Combined with Posterior Apophyseal Ring Separation in a Young Child: A Case Report. *Korean J Spine, 12*(3), 143–145. https://doi.org/10.14245/kjs.2015.12.3.143
328. Koehler, S. M., Rosario-Quinones, F., Mayer, J., McAnany, S., Schiller, A. L., Qureshi, S. & Hecht, A. C. (2014). Understanding acute apophyseal spinous process avulsion injuries. *Orthopedics, 37*(3), e317-21. https://doi.org/10.3928/01477447-20140225-68
329. Koptan, W. M. T., Elmiligui, Y. H. & Elsharkawi, M. M. (2011). Direct repair of spondylolysis presenting after correction of adolescent idiopathic scoliosis. *Spine Journal, 11*(2), 133–138. https://doi.org/10.1016/j.spinee.2011.01.012
330. Kozlowski, K. (1977). Anterior intervertebral disc herniations in children. Report of four cases. *Pediatr Radiol, 6*(1), 32–35.
331. Kozlowski, K. (1978). Anterior intervertebral disc herniations. (Report of six cases). *Fortschritte auf dem Gebiete der Röntgenstrahlen und der Nuklearmedizin, 129*(1), 47–49.
332. Kruppa, C. G., Khoriaty, J. D., Sietsema, D. L., Dudda, M., Schildhauer, T. A. & Jones, C. B. (2016). Pediatric pelvic ring injuries: How benign are they? *Injury, 47*(10), 2228–2234. https://doi.org/10.1016/j.injury.2016.07.002
333. Kuh, S. U., Kim, Y. S., Cho, Y. E., Yoon, Y. S., Jin, B. H., Kim, K. S. & Chin, D. K. (2005). Surgical treatments for lumbar disc disease in adolescent patients; chemonucleolysis/microsurgical discectomy/PLIF with cages. *Yonsei Medical Journal, 46*(1), 125–132.
334. Ladenhauf, H. N., Fabricant, P. D., Grossman, E., Widmann, R. F. & Green, D. W. (2013). Athletic participation in children with symptomatic spondylolysis in the New York Area. *Medicine and Science in Sports and Exercise, 45*(10), 1971–1974. https://doi.org/10.1249/MSS.0b013e318294b4ed
335. Lagerbäck, T., Elkan, P., Möller, H., Grauers, A., Diarbakerli, E. & Gerdhem, P. (2015). An observational study on the outcome after surgery for lumbar disc herniation in adolescents compared with adults based on the Swedish Spine Register. *Spine Journal, 15*(6), 1241–1247. https://doi.org/10.1016/j.spinee.2015.02.024
336. Landman, Z., Oswald, T., Sanders, J. & Diab, M. (2011). Prevalence and predictors of pain in surgical treatment of adolescent idiopathic scoliosis. *Spine (Phila Pa 1976), 36*(10), 825–829. https://doi.org/10.1097/BRS.0b013e3181de8c2b
337. Large, D. F., Doig, W. G., Dickens, D. R., Torode, I. P. & Cole, W. G. (1991). Surgical treatment of double major scoliosis. Improvement of the lumbar curve after fusion of the thoracic curve. *Journal of Bone and Joint Surgery - Series B, 73*(1), 121–124
338. Lemire, J. J., Mierau, D. R., Crawford, C. M. & Dzus, A. K. (1996). Scheuermann's juvenile kyphosis. *Journal of Manipulative and Physiological Therapeutics, 19*(3), 195–201
339. Leroux, J., Vivier, P. H., Ould Slimane, M., Foulongne, E., Abu-Amara, S., Lechevallier, J. & Griffet, J. (2013). Early diagnosis of thoracolumbar spine fractures in children. A prospective study. *Orthopaedics and Traumatology: Surgery and Research, 99*(1), 60–65. https://doi.org/10.1016/j.otsr.2012.10.009
340. Lundin, D. A., Wiseman, D., Ellenbogen, R. G. & Shaffrey, C. I. (2003). Direct repair of the pars interarticularis for spondylolysis and spondylolisthesis. *Pediatr Neurosurg, 39*(4), 195–200
341. Makino, T., Kaito, T., Kashii, M., Iwasaki, M. & Yoshikawa, H. (2015). Low back pain and patient-reported QOL outcomes in patients with adolescent idiopathic scoliosis without corrective surgery. *SpringerPlus, 4*(1), 397. https://doi.org/10.1186/s40064-015-1189-y
342. Marhaug, G. (1993). Idiopathic juvenile osteoporosis. *Scand J Rheumatol, 22*(1), 45–47.
343. Martin, J., Brandser, E. A., Shin, M. J. & Buckwalter, J. A. (1995). Fatigue fracture of the sacrum in a child. *Canadian Association of Radiologists Journal, 46*(6), 468–470.
344. Martin, R. P., Deane, R. H. & Collett, V. (1997). Spondylolysis in children who have osteopetrosis. *J Bone Joint Surg Am, 79*(11), 1685–1689
345. McCall, I. W., Park, W. M., O'Brien, J. P. & Seal, V. (1985). Acute traumatic intraosseous disc herniation. *Spine (Phila Pa 1976), 10*(2), 134–137.
346. Mehdian, S. M., Arun, R., Jones, A. & Cole, A. A. (2005). Reduction of severe adolescent isthmic spondylolisthesis: a new technique. *Spine (Phila Pa 1976), 30*(19), E579-84.

347. Melchior, R., Zabel, B., Spranger, J. & Schumacher, R. (2005). Effective parenteral clodronate treatment of a child with severe juvenile idiopathic osteoporosis. *Eur J Pediatr, 164*(1), 22–27. https://doi.org/10.1007/s00431-004-1541-7
348. Merola, A. A., Haher, T. R., Brkaric, M., Panagopoulos, G., Mathur, S., Kohani, O., Lowe, T. G., Lenke, L. G., Wenger, D. R., Newton, P. O., Clements Iii, D. H. & Betz, R. R. (2002). A multicenter study of the outcomes of the surgical treatment of adolescent idiopathic scoliosis using the Scoliosis Research Society (SRS) outcome instrument. *Spine, 27*(18), 2046–2051. https://doi.org/10.1097/00007632-200209150-00015
349. Micheli, L. J. & Wood, R. (1995). Back pain in young athletes. Significant differences from adults in causes and patterns. *Arch Pediatr Adolesc Med, 149*(1), 15–18
350. Miyagi, R., Sairyo, K., Sakai, T., Tezuka, F., Kitagawa, Y. & Dezawa, A. (2014). Persistent tight hamstrings following conservative treatment for apophyseal ring fracture in adolescent athletes: Critical appraisal. *Journal of Medical Investigation, 61*(3-4), 446–451. https://doi.org/10.2152/jmi.61.446
351. Miyakoshi, N., Kobayashi, A., Hongo, M. & Shimada, Y. (2015). Sacral rib: an uncommon congenital anomaly. *Spine J, 15*(6), e35-8. https://doi.org/10.1016/j.spinee.2013.08.055
352. Molina, V., Court, C., Dagher, G., Pourjamasb, B. & Nordin, J. Y. (2004). Fracture of the posterior margin of the lumbar spine: case report after an acute, unique, and severe trauma. *Spine (Phila Pa 1976), 29*(24), E565-7.
353. Moller, A., Hasserius, R., Besjakov, J., Ohlin, A. & Karlsson, M. (2006). Vertebral fractures in late adolescence: A 27 to 47-year follow-up. *European Spine Journal, 15*(8), 1247–1254. https://doi.org/10.1007/s00586-005-0043-2
354. Mounasamy, V., Myers, B. & Phillips, J. H. (2006). Ganglion cyst of a lumbar facet joint in an adolescent - A case report. *European Journal of Orthopaedic Surgery and Traumatology, 16*(3), 231–233. https://doi.org/10.1007/s00590-005-0063-y
355. Negrini, S., Minozzi, S., Bettany-Saltikov, J., Chockalingam, N., Grivas, T. B., Kotwicki, T., Maruyama, T., Romano, M. & Zaina, F. (2015). Braces for idiopathic scoliosis in adolescents. *Cochrane Database of Systematic Reviews*(6)
356. Obukhov, S. K., Hankenson, L., Manka, M. & Mawk, J. R. (1996). Multilevel lumbar disc herniation in 12-year-old twins. *Child's Nervous System, 12*(3), 169–171. https://doi.org/10.1007/BF00266823
357. Ozgen, S., Konya, D., Toktas, O. Z., Dagcinar, A. & Ozek, M. M. (2007). Lumbar disc herniation in adolescence. *Pediatr Neurosurg, 43*(2), 77–81. https://doi.org/10.1159/000098377
358. Parisini, P., Di Silvestre, M., Greggi, T., Miglietta, A. & Paderni, S. (2001). Lumbar disc excision in children and adolescents. *Spine (Phila Pa 1976), 26*(18), 1997–2000.
359. Peh, W. C. G., Griffith, J. F., Yip, D. K. H. & Leong, J. C. Y. (1998). Magnetic resonance imaging of lumbar vertebral apophyseal ring fractures. *Australasian Radiology, 42*(1), 34–37.
360. Pinto, F. C., Poetscher, A. W., Quinhones, F. R., Pena, M. & Taricco, M. A. (2002). Lumbar disc herniation associated with scoliosis in a 15-year-old girl: case report. *Arq Neuropsiquiatr, 60*(2-a), 295–298.
361. Piper, S. & Degraauw, C. (2012). A 14-year-old competitive, high-level athlete with unilateral low back pain: case report. *J Can Chiropr Assoc, 56*(4), 283–291.
362. Polly, D. W., Jr. & Mason, D. E. (1991). Congenital absence of a lumbar pedicle presenting as back pain in children. *Journal of Pediatric Orthopaedics, 11*(2), 214–219.
363. Popko, J., Konstantynowicz, J., Kossakowski, D., Kaczmarski, M. & Piotrowska-Jastrzebska, J. (1997). Assessment of bone density in children with Scheuermann's disease. *Rocz Akad Med Bialymst, 42*(1), 245–250
364. Posch, E., Schwarz, N., Fischmeister, F. M., Mayr, J. & Schwarz, A. F. (1998). Unstable pelvic ring fractures. *Acta Chirurgica Austriaca, 30*(SUPPL. 143), 52–54.
365. Quinlan, E., Reinke, T. & Bogar, W. C. (2013). Spinous process apophysitis: a cause of low back pain following repetitive hyperextension in an adolescent female dancer. *J Dance Med Sci, 17*(4), 170–174.
366. Ralston, S. & Weir, M. (1998). Suspecting lumbar spondylolysis in adolescent low back pain. *Clin Pediatr (Phila), 37*(5), 287–293.
367. Ramirez, N., Johnston, C. E. & Browne, R. H. (1997). The prevalence of back pain in children who have idiopathic scoliosis. *J Bone Joint Surg Am, 79*(3), 364–368.
368. Raudenbush, B. L., Chambers, R. C., Silverstein, M. P. & Goodwin, R. C. (2017). Indirect pars repair for pediatric isthmic spondylolysis: a case series. *J Spine Surg, 3*(3), 387–391. https://doi.org/10.21037/jss.2017.08.08
369. Read, M. T. (1994). Single photon emission computed tomography (SPECT) scanning for adolescent back pain. A sine qua non? *Br J Sports Med, 28*(1), 56–57
370. Rodd, C., Lang, B., Ramsay, T., Alos, N., Huber, A. M., Cabral, D. A., Scuccimarri, R., Miettunen, P. M., Roth, J., Atkinson, S. A., Couch, R., Cummings, E. A., Dent, P. B., Ellsworth, J., Hay, J., Houghton, K., Jurencak, R., Larché, M., LeBlanc, C., . . . Ward, L. M. (2012). Incident vertebral fractures among children with rheumatic disorders 12 months after glucocorticoid initiation: A national observational study. *Arthritis Care and Research, 64*(1), 122–131. https://doi.org/10.1002/acr.20589
371. Rodríguez, B. M., Sánchez, R. F., Abellán, E. D., Parra, J. Z., Canovas, C. S. & Sánchez, M. I. C. (2015). Bertolotti syndrome: A little known cause of low-back pain in childhood. *Journal of Pediatrics, 166*(1), 202-202.e1. https://doi.org/10.1016/j.jpeds.2014.08.036
372. Rysavy, M., Khayarin, M. A. & Arun, K. (2003). Sacroiliac joint dislocation in 11 years old boy treated by open reduction and internal fixation. *Acta Chir Orthop Traumatol Cech, 70*(2), 112–115.

373. Sakai, T., Goda, Y., Tezuka, F., Takata, Y., Higashino, K., Sato, M., Mase, Y., Nagamachi, A. & Sairyo, K. (2016). Characteristics of lumbar spondylolysis in elementary school age children. *Eur Spine J, 25*(2), 602–606. https://doi.org/10.1007/s00586-015-4029-4
374. Sato, T., Hirano, T., Ito, T., Morita, O., Kikuchi, R., Endo, N. & Tanabe, N. (2011). Back pain in adolescents with idiopathic scoliosis: Epidemiological study for 43,630 pupils in Niigata City, Japan. *European Spine Journal, 20*(2), 274–279. https://doi.org/10.1007/s00586-010-1657-6
375. Sattar, T., Bannister, C. M. & Turnbull, L. W. (1997). Long term outcome of 83 patients with occult spinal dysraphsm. *European Journal of Pediatric Surgery, Supplement, 7*(1), 40
376. Sbrocchi, A. M., Rauch, F., Matzinger, M., Feber, J. & Ward, L. M. (2011). Vertebral fractures despite normal spine bone mineral density in a boy with nephrotic syndrome. *Pediatr Nephrol, 26*(1), 139–142. https://doi.org/10.1007/s00467-010-1652-5
377. Schwarz, N., Mayr, J., Fischmeister, F. M., Schwarz, A. F., Posch, E. & Ohner, T. (1994) [2 years results of conservative therapy of unstable fractures of the pelvic ring in children]. *Unfallchirurg, 97*(9), 439–444. (Erstveröffentlichung 2-Jahres-Ergebnisse der konservativen Therapie instabiler Beckenringfrakturen bei Kindern.)
378. Schwarz, N., Posch, E., Mayr, J., Fischmeister, F. M., Schwarz, A. F. & Ohner, T. (1998). Long-term results of unstable pelvic ring fractures in children. *Injury, 29*(6), 431–433.
379. Selhorst, M., Fischer, A., Graft, K., Ravindran, R., Peters, E., Rodenberg, R. & MacDonald, J. (2016). Long-Term Clinical Outcomes and Factors That Predict Poor Prognosis in Athletes After a Diagnosis of Acute Spondylolysis: A Retrospective Review With Telephone Follow-up. *J Orthop Sports Phys Ther, 46*(12), 1029–1036. https://doi.org/10.2519/jospt.2016.7028
380. Selhorst, M., Fischer, A. & MacDonald, J. (2019). Prevalence of spondylolysis in symptomatic adolescent athletes: an assessment of sport risk in nonelite athletes. *Clin J Sport Med, 29*(5), 421–425
381. Semeao, E. J., Stallings, V. A., Peck, S. N. & Piccoli, D. A. (1997). Vertebral compression fractures in pediatric patients with Crohn's disease. *Gastroenterology, 112*(5), 1710–1713.
382. Siebens, A. A., Hungerford, D. S. & Kirby, N. A. (1987). Achondroplasia: Effectiveness of an orthosis in reducing deformity of the spine. *Archives of Physical Medicine and Rehabilitation, 68*(6), 384–388
383. Smith, R. (1980). Idiopathic osteoporosis in the young. *Journal of Bone and Joint Surgery - Series B, 62*(4), 417–427.
384. Smith, R. (1995). Idiopathic juvenile osteoporosis: Experience of twenty-one patients. *Rheumatology, 34*(1), 68–77. https://doi.org/10.1093/rheumatology/34.1.68
385. Smorgick, Y., Floman, Y., Millgram, M. A., Anekstein, Y., Pekarsky, I. & Mirovsky, Y. (2006). Mid- to long-term outcome of disc excision in adolescent disc herniation. *Spine Journal, 6*(4), 380–384. https://doi.org/10.1016/j.spinee.2005.10.015
386. Soliman, H. M. (2016). Irrigation endoscopic assisted percutaneous pars repair: Technical note. *Spine J, 16*(10), 1276–1281. https://doi.org/10.1016/j.spinee.2016.06.009
387. Solomou, A., Kraniotis, P., Rigopoulou, A. & Petsas, T. (2018). Frequent Benign, Nontraumatic, Noninflammatory Causes of Low Back Pain in Adolescents: MRI Findings. *Radiol Res Pract*, 2018, 7638505. https://doi.org/10.1155/2018/7638505
388. Sousa, T., Skaggs, D. L., Chan, P., Yamaguchi, K. T., Jr., Borgella, J., Lee, C., Sawyer, J., Moisan, A., Flynn, J. M., Gunderson, M., Hresko, M. T., D'Hemecourt, P. & Andras, L. M. (2017). Benign Natural History of Spondylolysis in Adolescence With Midterm Follow-Up. *Spine Deformity, 5*(2), 134–138. https://doi.org/10.1016/j.jspd.2016.10.005
389. Spapens, N., Wouters, C. & Moens, P. (2010). Thoracolumbar intervertebral disc calcifications in an 8-year-old boy: Case report and review of the literature. *European Journal of Pediatrics, 169*(5), 577–580. https://doi.org/10.1007/s00431-009-1076-z
390. Stäbler, A., Paulus, R., Steinborn, M., Bosch, R., Matzko, M. & Reiser, M. (2000) [Spondylolysis in the developmental stage diagnostic contribution of MRI]. *Röfo, 172*(1), 33–37. https://doi.org/10.1055/s-2000-278 (Erstveröffentlichung Die Spondylolyse im Stadium der Entstehung: Diagnostischer Beitrag der MRT
391. Subasi, M., Arslan, H., Necmioglu, S., Onen, A., Ozen, S. & Kaya, M. (2004). Long-term outcomes of conservatively treated paediatric pelvic fractures. *Injury, 35*(8), 771–781. https://doi.org/10.1016/j.injury.2003.09.037
392. Sumita, T., Sairyo, K., Shibuya, I., Kitahama, Y., Kanamori, Y., Matsumoto, H., Koga, S., Kitagawa, Y. & Dezawa, A. (2013). V-Rod technique for direct repair surgery of pediatric lumbar spondylolysis combined with posterior apophyseal ring fracture. *Asian Spine Journal, 7*(2), 115–118. https://doi.org/10.4184/asj.2013.7.2.115
393. Sutton, T. J. & Turcotte, B. (1973). Posterior herniation of calcified intervertebral discs in children. *Canadian Association of Radiologists Journal, 24*(2), 131–136.
394. Swierkosz, S. & Nowak, Z. (2015). Low back pain in adolescents. An assessment of the quality of life in terms of qualitative and quantitative pain variables. *J Back Musculoskelet Rehabil, 28*(1), 25–34. https://doi.org/10.3233/bmr-140484
395. Swischuk, L. E. & Stansberry, S. D. (1991). Calcific discitis: MRI changes in discs without visible calcification. *Pediatric Radiology, 21*(5), 365–366. https://doi.org/10.1007/BF02011490
396. Takahashi, Y., Kobayashi, T., Miyakoshi, N., Abe, E., Abe, T., Kikuchi, K. & Shimada, Y. (2016). Sacral stress fracture in an amateur rugby player: a case report. *J Med Case Rep, 10*(1), 327. https://doi.org/10.1186/s13256-016-1120-3
397. Tamaki, S., Yamashita, K., Higashino, K., Sakai, T., Takata, Y. & Sairyo, K. (2016). Lumbar Posterior Apophyseal Ring Fracture Combined with Spondylolysis in Pediatric Athletes: A Report of Three Cases. *JBJS Case Connector, 6*(3), e64. https://doi.org/10.2106/JBJS.CC.15.00245
398. Tan, L. O., Lim, S. Y. & Vasanwala, R. F. (2017). Primary osteoporosis in children. *BMJ Case Rep*, 2017. https://doi.org/10.1136/bcr-2017-220700

399. Taylor, G. A. & Eggli, K. D. (1988). Lap-belt injuries of the lumbar spine in children: a pitfall in CT diagnosis. *AJR Am J Roentgenol, 150*(6), 1355–1358. https://doi.org/10.2214/ajr.150.6.1355
400. Théroux, J., May, S. L., Fortin, C. & Labelle, H. (2015). Prevalence and management of back pain in adolescent idiopathic scoliosis patients: A retrospective study. *Pain Research and Management, 20*(3), 153–157.
401. Théroux, J., Stomski, N., Hodgetts, C. J., Ballard, A., Khadra, C., Le May, S. & Labelle, H. (2017). Prevalence of low back pain in adolescents with idiopathic scoliosis: A systematic review. *Chiropractic and Manual Therapies, 25*(1). https://doi.org/10.1186/s12998-017-0143-1
402. Toto, B. & Shapiro, I. (1995). Diagnosis and treatment of spondylolysis in an adolescent athlete. *Chiropractic Sports Medicine, 9*(3), 100–105
403. Ueda, Y., Kawahara, N., Murakami, H., Demura, S. & Tsuchiya, H. (2012). Thoracic disk herniation with paraparesis treated with transthoracic microdiskectomy in a 14-year-old girl. *Orthopedics, 35*(5), e774-7. https://doi.org/10.3928/01477447-20120426-41
404. van Buskirk, C. S. & Ritterbusch, J. F. (1997). Natural history of distal spinal agenesis. *J Pediatr Orthop B, 6*(2), 146–152
405. Vrable, A. & Sherman, A. L. (2009). Elite male adolescent gymnast who achieved union of a persistent bilateral pars defect. *Am J Phys Med Rehabil, 88*(2), 156–160. https://doi.org/10.1097/PHM.0b013e31819515c0
406. Wang, H., Cheng, J., Xiao, H., Li, C. & Zhou, Y. (2013). Adolescent lumbar disc herniation: Experience from a large minimally invasive treatment centre for lumbar degenerative disease in Chongqing, China. *Clinical Neurology and Neurosurgery, 115*(8), 1415–1419. https://doi.org/10.1016/j.clineuro.2013.01.019
407. Wasowska-Krolikowska, K. & Krogulska, A. (1998). Idiopathic juvenile osteoporosis - Observation in the course of several years. *Medical Science Monitor, 4*(6), 1075–1079.
408. Weir, M. R. & Smith, D. S. (1989). Stress reaction of the pars interarticularis leading to spondylolysis. A cause of adolescent low back pain. *J Adolesc Health Care, 10*(6), 573–577.
409. Wessely, M. A., Mick, T. J. & Brandt, J. (2010). Low back pain in an adolescent American Footballer: Case discussion. *Clinical Chiropractic, 13*(1), 135–140. https://doi.org/10.1016/j.clch.2010.02.013
410. Wilson, F. D. & Lindseth, R. E. (1982). The adolescent "swimmer's back". *Am J Sports Med, 10*(3), 174–176
411. Yen, C. H., Chan, S. K., Ho, Y. F. & Mak, K. H. (2009). Posterior lumbar apophyseal ring fractures in adolescents: a report of four cases. *J Orthop Surg (Hong Kong), 17*(1), 85–89.
412. Zamani, M. H. & MacEwen, G. D. (1982). Herniation of the lumbar disc in children and adolescents. *J Pediatr Orthop, 2*(5), 528–533.
413. Zapata, K. A., Wang-Price, S. S., Sucato, D. J., Thompson, M., Trudelle-Jackson, E. & Lovelace-Chandler, V. (2015). Spinal Stabilization Exercise Effectiveness for Low Back Pain in Adolescent Idiopathic Scoliosis: A Randomized Trial. *Pediatric Physical Therapy, 27*(4), 396–402. https://doi.org/10.1097/PEP.0000000000000174
414. Zhang, W., Kaplan, S. L., Servaes, S. & Zhuang, H. (2015). Limbus Vertebra on Bone Scintigraphy in a Pediatric Patient. *Clin Nucl Med, 40*(11), 915–916. https://doi.org/10.1097/rlu.0000000000000970

Neurological and neuromuscular diseases

415. Adams, C. & Armstrong, D. (1990). Acute Transverse Myelopathy in Children. *Canadian Journal of Neurological Sciences / Journal Canadien des Sciences Neurologiques, 17*(1), 40–45. https://doi.org/10.1017/S0317167100030006
416. Azumagawa, K., Yamamoto, S., Tanaka, K., Sakanaka, H., Teraura, H., Takahashi, K. & Tamai, H. (2012). Non-operative treated spontaneous spinal epidural hematoma in a 12-year-old boy. *Pediatric Emergency Care, 28*(2), 167–169. https://doi.org/10.1097/PEC.0b013e318244785d
417. Begeer, J. H., Meihuizen de Regt, M. J., HogenEsch, I., Ter Weeme, C. A., Mooij, J. J. & Vencken, L. M. (1986). Progressive neurological deficit in children with spina bifida aperta. *Z Kinderchir, 41* Suppl 1, 13–15. https://doi.org/10.1055/s-2008-1043387
418. Bond, A. E., Zada, G., Bowen, I., McComb, J. G. & Krieger, M. D. (2012). Spinal arachnoid cysts in the pediatric population: report of 31 cases and a review of the literature. *J Neurosurg Pediatr, 9*(4), 432–441. https://doi.org/10.3171/2012.1.peds11391
419. Chen, A. M., Neustadt, J. B. & Kucera, J. N. (2017). Rib head dislocation causing spinal canal stenosis in a child with neurofibromatosis, type 1. *J Radiol Case Rep, 11*(8), 8–15. https://doi.org/10.3941/jrcr.v11i8.3113
420. Colak, A., Pollack, I. F. & Albright, A. L. (1998). Recurrent tethering: A common long-term problem after lipomyelomeningocele repair. *Pediatric Neurosurgery, 29*(4), 184–190. https://doi.org/10.1159/000028719
421. Davis, G. A. & Klug, G. L. (2000). Acute-onset nontraumatic paraplegia in childhood: Fibrocartilaginous embolism or acute myelitis? *Child's Nervous System, 16*(9), 551–554.
422. Dillen, W. L., Hendricks, B. K., Mannas, J. P. & Wheeler, G. R. (2018). Surfer's myelopathy: A rare presentation in a teenage gymnast and review of the literature. *J Clin Neurosci, 50*, 157–160. https://doi.org/10.1016/j.jocn.2018.01.039

423. Doralp, S. & Bartlett, D. J. (2010). The prevalence, distribution, and effect of pain among adolescents with cerebral palsy. *Pediatric Physical Therapy, 22*(1), 26–33. https://doi.org/10.1097/PEP.0b013e3181ccbabb
424. Dunne, K., Hopkins, I. J. & Shield, L. K. (1986). ACUTE TRANSVERSE MYELOPATHY IN CHILDHOOD. *Developmental Medicine & Child Neurology, 28*(2), 198–204. https://doi.org/10.1111/j.1469-8749.1986.tb03855.x
425. Dure, L. S., Percy, A. K., Cheek, W. R. & Laurent, J. P. (1989). Chiari type I malformation in children. *J Pediatr, 115*(4), 573–576.
426. Eid, R., Raj, A., Farber, D., Puri, V. & Bertolone, S. (2016). Spinal cord infarction in hemoglobin SC disease as an amusement park accident. *Pediatrics, 138*(3). https://doi.org/10.1542/peds.2015-4020
427. Evangelou, P., Meixensberger, J., Bernhard, M., Hirsch, W., Kiess, W., Merkenschlager, A., Nestler, U. & Preuss, M. (2013). Operative management of idiopathic spinal intradural arachnoid cysts in children: A systematic review. *Child's Nervous System, 29*(4), 657–664. https://doi.org/10.1007/s00381-012-1990-7
428. Foreman, P., Safavi-A Bbasi, S., Talley, M. C., Boeckman, L. & Mapstone, T. B. (2012). Perioperative outcomes and complications associated with allogeneic duraplasty for the management of Chiari malformations Type i in 48 pediatric patients: Clinical article. Journal of Neurosurgery: *Pediatrics, 10*(2), 142–149. https://doi.org/10.3171/2012.5.PEDS11406
429. Gennari, J. M., Themar-Noel, C., Panuel, M., Bensamoun, B., Deslandre, C., Linglart, A., Sokolowski, M. & Ferrari, A. (2015). Adolescent spinal pain: The pediatric orthopedist's point of view. *Orthopaedics and Traumatology: Surgery and Research, 101*(6), S247-S250. https://doi.org/10.1016/j.otsr.2015.06.012
430. Geyik, M., Alptekin, M., Erkutlu, I., Geyik, S., Erbas, C., Pusat, S. & Kural, C. (2015). Tethered cord syndrome in children: a single-center experience with 162 patients. *Childs Nerv Syst, 31*(9), 1559–1563. https://doi.org/10.1007/s00381-015-2748-9
431. Hoffman, H. J., Hendrick, E. B. & Humphreys, R. P. (1976). The tethered spinal cord: Its protean manifestations, diagnosis and surgical correction. *Pediatric Neurosurgery, 2*(3), 145–155. https://doi.org/10.1159/000119610
432. Hsu, P. C. & Chen, S. J. (2017). Longitudinal extensive transverse myelitis with an abnormal uFLC ratio in a pediatric patient: Case report and literature review. *Medicine (Baltimore), 96*(52), e9389. https://doi.org/10.1097/md.0000000000009389
433. Joseph, R. N., Batty, R., Raghavan, A., Sinha, S., Griffiths, P. D. & Connolly, D. J. A. (2013). Management of isolated syringomyelia in the paediatric population-a review of imaging and follow-up in a single centre. *British Journal of Neurosurgery, 27*(5), 683–686. https://doi.org/10.3109/02688697.2013.771728
434. Kawecki, Z., Fafara, A., Kwiatkowski, S., Maryńczak, L., Milczarek, O., Kwiatkowski, T., Herman-Sucharska, I. & Wojtak, J. (2011). Tethered cord syndrome in children. *Journal of Orthopaedics Trauma Surgery and Related Research*(21), 39–48.
435. Komarowska, M., Debek, W., Wojnar, J. A., Hermanowicz, A. & Rogalski, M. (2013). Brown-Séquard syndrome in a 11-year-old girl due to penetrating glass injury to the thoracic spine. *European Journal of Orthopaedic Surgery and Traumatology, 23*(SUPPL. 2), S141-S143. https://doi.org/10.1007/s00590-012-1050-8
436. Kulwin, C. G., Patel, N. B., Ackerman, L. L., Smith, J. L., Boaz, J. C. & Fulkerson, D. H. (2013). Radiographic and clinical outcome of syringomyelia in patients treated for tethered cord syndrome without other significant imaging abnormalities. *Journal of Neurosurgery: Pediatrics, 11*(3), 307–312. https://doi.org/10.3171/2012.11.PEDS12251
437. Lannum, S. & Stratton, J. (2009). Spontaneous epidural hematoma of the thoracic spine in a 17-year-old adolescent boy: a case report. *Am J Emerg Med, 27*(5), 628.e5-6. https://doi.org/10.1016/j.ajem.2008.08.031
438. Lee, K. S. (1996). Delayed central cord syndrome after a handstand in a child: Case report. *Spinal Cord, 34*(3), 176–178.
439. Li, P. H., Chang, H. S., Huang, H. Y. & Lin, J. S. (2000). Guillain-Barre syndrome presenting with severe pain: Report of one case. *Acta Paediatrica Taiwanica, 41*(1), 33–35.
440. Lohani, S., Robertson, R. L. & Proctor, M. R. (2013). Ruptured temporal lobe arachnoid cyst presenting with severe back pain. *J Neurosurg Pediatr, 12*(3), 281–283. https://doi.org/10.3171/2013.6.peds13122
441. Lundkvist, K., Cson Silander, H., Dahl, M. & Stromberg, B. (1997). Tethered cord release: A 10 war retrospective study. *European Journal of Pediatric Surgery, Supplement, 7*(1), 11.
442. Martin-Fuentes, A. M., Pretell-Mazzini, J., Curto de la Mano, A. & Vina-Fernandez, R. (2013). High-grade spondyloretrolisthesis in a 12-year-old girl with neurofibromatosis type 1: a case report and literature review. *J Pediatr Orthop B, 22*(2), 110–116. https://doi.org/10.1097/BPB.0b013e328357eac2
443. Miró, J., La Vega, R. de, Tomé-Pires, C., Sánchez-Rodríguez, E., Castarlenas, E., Jensen, M. P. & Engel, J. M. (2017). Pain extent and function in youth with physical disabilities. *Journal of Pain Research, 10*, 113–120. https://doi.org/10.2147/JPR.S121590
444. Nazar, G. B., Casale, A. J., Roberts, J. G. & Linden, R. D. (1995). Occult filum terminale syndrome. *Pediatric Neurosurgery, 23*(5), 228–235. https://doi.org/10.1159/000120965
445. Nguyen, D. K., Agenarioti-Bélanger, S. & Vanasse, M. (1999). Pain and the Guillain-Barre syndrome in children under 6 years old. *Journal of Pediatrics, 134*(6), 773–776.
446. Ogiwara, H., Lyszczarz, A., Alden, T. D., Bowman, R. M., McLone, D. G. & Tomita, T. (2011). Retethering of transected fatty filum terminales. *J Neurosurg Pediatr, 7*(1), 42–46. https://doi.org/10.3171/2010.10.peds09550
447. Ostling, L. R., Bierbrauer, K. S. & Kuntz, C. t. (2012). Outcome, reoperation, and complications in 99 consecutive children operated for tight or fatty filum. *World Neurosurg, 77*(1), 187–191. https://doi.org/10.1016/j.wneu.2011.05.017

448. Park, D. H., Cho, T. H., Lee, J. B., Park, J. Y., Park, Y. K., Chung, Y. G. & Suh, J. K. (2008). Rapid spontaneous remission of a spontaneous spinal chronic subdural hematoma in a child - Case report. *Neurologia Medico-Chirurgica, 48*(5), 231–234. https://doi.org/10.2176/nmc.48.231
449. Robertson, W. C., Jr., Lee, Y. E. & Bruce Edmonson, M. (1979). Spontaneous spinal epidural hematoma in the young. *Neurology, 29*(1), 120–122.
450. Rodriguez, A., Kuhn, E. N., Somasundaram, A. & Couture, D. E. (2015). Management of idiopathic pediatric syringohydromyelia. *J Neurosurg Pediatr, 16*(4), 452–457. https://doi.org/10.3171/2015.3.peds14433
451. Rosenberg, O., Itshayek, E. & Israel, Z. (2003). Spontaneous spinal epidural hematoma in a 14-year-old girl: Case report and review of the literature. *Pediatric Neurosurgery, 38*(4), 216–218. https://doi.org/10.1159/000069091
452. Sano, H., Satomi, K. & Hirano, J. (2004). Recurrent idiopathic epidural hematoma: A case report. *Journal of Orthopaedic Science, 9*(6), 625–628. https://doi.org/10.1007/s00776-004-0821-4
453. Srinivas, H. & Kumar, A. (2014). Silent neurenteric cyst with split cord malformation at conus medullaris: Case report and literature review. *J Pediatr Neurosci, 9*(3), 246–248. https://doi.org/10.4103/1817-1745.147579
454. Stanitski, C. L., Micheli, L. J., Hall, J. E. & Rosenthal, R. K. (1982). Surgical correction of spinal deformity in cerebral palsy. *Spine (Phila Pa 1976), 7*(6), 563–569.
455. Tailor, J., Dunn, I. F. & Smith, E. (2006). Conservative treatment of spontaneous spinal epidural hematoma associated with oral anticoagulant therapy in a child. *Childs Nerv Syst, 22*(12), 1643–1645. https://doi.org/10.1007/s00381-006-0220-6
456. Tewari, M. K., Tripathi, L. N., Mathuriya, S. N., Khandelwal, N. & Kak, V. K. (1992). Spontaneous spinal extradural hematoma in children - Report of three cases and a review of the literature. *Child's Nervous System, 8*(1), 53–55. https://doi.org/10.1007/BF00316564
457. Tyagi, R., Kloepping, C. & Shah, S. (2016). Spinal cord stimulation for recurrent tethered cord syndrome in a pediatric patient: case report. *J Neurosurg Pediatr, 18*(1), 105–110. https://doi.org/10.3171/2015.12.peds14645
458. Wehby, M. C., O'Hollaren, P. S., Abtin, K., Hume, J. L. & Richards, B. J. (2004). Occult tight filum terminale syndrome: Results of surgical untethering. *Pediatric Neurosurgery, 40*(2), 51–57. https://doi.org/10.1159/000078908
459. Weissert, M., Gysler, R. & Sorensen, N. (1989) [The clinical problem of the tethered cord syndrome--a report of 3 personal cases]. *Z Kinderchir, 44*(5), 275–279. https://doi.org/10.1055/s-2008-1043250 (Erstveröffentlichung Zur klinischen Problematik des Tethered-Cord-Syndroms--Bericht uber drei eigene Beobachtungen.)
460. Wilmshurst, J. M., Thomas, N. H., Robinson, R. O., Bingham, J. B. & Pohl, K. R. E. (2001). Lower limb and back pain in Guillain-Barré syndrome and associated contrast enhancement in MRI of the cauda equina. *Acta Paediatrica, International Journal of Paediatrics, 90*(6), 691–694.
461. Zhou, Y., Zhu, L., Lin, Y. & Cheng, H. (2017). Chiari type i malformation with occult tethered cord syndrome in a child: A case report. *Medicine (United States), 96*(40). https://doi.org/10.1097/MD.0000000000008239

Rheumatic and inflammatory diseases

462. Aydin, F., Ozcakar, Z. B., Cakar, N., Celikel, E., Uncu, N., Celikel Acar, B. & Yalcinkaya, F. (2018). Sacroiliitis in Children With Familial Mediterranean Fever. *J Clin Rheumatol.* Epub ahead of print. https://doi.org/10.1097/rhu.0000000000000770
463. Binnetoğlu, K. K., Karakoç Aydiner, E., Bariş, S., Özen, A., Zöhre, A. İ., Baltacioğlu, F., Direskeneli, H. & Barlan, I. (2015). Atypical presentation of takayasu's arteritis in an adolescent. *Marmara Medical Journal, 28*(3), 157–160. https://doi.org/10.5472/MMJcr.2803.01
464. Bollow, M., Biedermann, T., Kannenberg, J., Paris, S., Schauer-Petrowski, C., Minden, K., Schöntube, M., Hamm, B., Sieper, J. & Braun, J. (1998). Use of dynamic magnetic resonance imaging to detect sacroiliitis in HLA- B27 positive and negative children with juvenile arthritides. *Journal of Rheumatology, 25*(3), 556–564.
465. Brown, R. T. (1981). Costochondritis in adolescents. *Journal of Adolescent Health Care, 1*(3), 198–201. https://doi.org/10.1016/S0197-0070(81)80056-3
466. Burgos-Vargas, R. (2009). A case of childhood-onset ankylosing spondylitis: Diagnosis and treatment. *Nature Clinical Practice Rheumatology, 5*(1), 52–57. https://doi.org/10.1038/ncprheum0971
467. Campos, T. A., Rebelo, J., Maia, A. & Brito, I. (2011). Chronic Recurrent Multifocal Osteomyelitis: An entity to Recognize! *Arquivos de Medicina, 25*(5-6), 183–185.
468. Demharter, J., Bohndorf, K., Michl, W. & Vogt, H. (1997). Chronic recurrent multifocal osteomyelitis: A radiological and clinical investigation of five cases. *Skeletal Radiology, 26*(10), 579–588. https://doi.org/10.1007/s002560050290
469. Duman, M. A., Duru, N. S., Caliskan, B., Sandikci, H. & Cengel, F. (2016). Lumbar Swelling as the Unusual Presentation of Henoch-Schonlein Purpura in a Child. *Balkan Med J, 33*(3), 360–362. https://doi.org/10.5152/balkanmedj.2016.150208
470. Fink, C. W. & Cimaz, R. G. (1995). Back pain as the presenting symptom in juvenile dermato/polymyositis. *J Clin Rheumatol, 1*(2), 90–92.

471. Fukumori, K., Shakado, S., Miyahara, T., Fukuizumi, K., Takemoto, R., Nishi, H., Sakai, H., Muranaka, T. & Sata, M. (2005). Atypical manifestations of pancreatitis with autoimmune phenomenon in an adolescent female. *Intern Med, 44*(8), 886–891.
472. Gemmel, F., Coningh, A. de, Collins, J. & Rijk, P. (2011). SPECT/CT of osteitis condensans ilii: one-stop shop imaging. *Clin Nucl Med, 36*(1), 59–61. https://doi.org/10.1097/RLU.0b013e3181feefe8
473. Girschick, H. J., Mornet, E., Beer, M., Warmuth-Metz, M. & Schneider, P. (2007). Chronic multifocal non-bacterial osteomyelitis in hypophosphatasia mimicking malignancy. *BMC Pediatr, 7*, 3. https://doi.org/10.1186/1471-2431-7-3
474. Girschick, H. J., Zimmer, C., Klaus, G., Darge, K., Dick, A. & Morbach, H. (2007). Chronic recurrent multifocal osteomyelitis: What is it and how should it be treated? *Nature Clinical Practice Rheumatology, 3*(12), 733–738. https://doi.org/10.1038/ncprheum0653
475. Haddock, G., Coupar, G., Youngson, G. G., MacKinlay, G. A. & Raine, P. A. M. (1994). Acute pancreatitis in children: A 15-year review. *Journal of Pediatric Surgery, 29*(6), 719–722. https://doi.org/10.1016/0022-3468(94)90353-0
476. Häfner, R. (1987). Juvenile spondarthritis - A retrospective study of 71 patients. *Monatsschrift fur Kinderheilkunde, 135*(1), 41–46.
477. Horneff, G., Fitter, S., Foeldvari, I., Minden, K., Kuemmerle-Deschner, J., Tzaribacev, N., Thon, A., Borte, M., Ganser, G., Trauzeddel, R. & Huppertz, H. I. (2012). Double-blind, placebo-controlled randomized trial with adalimumab for treatment of juvenile onset ankylosing spondylitis (JoAS): Significant short term improvement. *Arthritis Res Ther, 14*(5), R230. https://doi.org/10.1186/ar4072
478. Kekilli, E., Yagmur, C. & Aydin, O. M. (2004). Cervical involvement in juvenile-onset ankylosing spondylitis with bone scintigraphy. *Rheumatology International, 24*(3), 164–165. https://doi.org/10.1007/s00296-003-0367-3
479. Moussa, T., Bhat, V., Kini, V. & Fathalla, B. M. (2016). Clinical and genetic association, radiological findings and response to biological therapy in seven children from Qatar with non-bacterial osteomyelitis. *Int J Rheum Dis*. Epub ahead of print. https://doi.org/10.1111/1756-185x.12940
480. Pagnini, I., Savelli, S., Matucci-Cerinic, M., Fonda, C., Cimaz, R. & Simonini, G. (2010). Early predictors of juvenile sacroiliitis in enthesitis-related arthritis. *J Rheumatol, 37*(11), 2395–2401. https://doi.org/10.3899/jrheum.100090
481. Park, J. H., Seo, Y. M., Han, S. B., Kim, K. H., Rhim, J. W., Chung, N. G., Kim, M. S., Kang, J. H. & Jeong, D. C. (2016). Recurrent macrophage activation syndrome since toddler age in an adolescent boy with HLA B27 positive juvenile ankylosing spondylitis. *Korean J Pediatr, 59*(10), 421–424. https://doi.org/10.3345/kjp.2016.59.10.421
482. Stoler, J., Biller, J. A. & Grand, R. J. (1987). Pancreatitis in Kawasaki Disease. *American Journal of Diseases of Children, 141*(3), 306–308. https://doi.org/10.1001/archpedi.1987.04460030084031
483. Stump, D., Spock, A. & Grossman, H. (1976). Vertebral sarcoidosis in adolescents. *Radiology, 121*(1), 153–155. https://doi.org/10.1148/121.1.153
484. Takase, M., Imai, T. & Nozaki, F. (2010). Relapsing autoimmune pancreatitis in a 14-year-old girl. *J Nippon Med Sch, 77*(1), 29–34.
485. Tateyama, T., Waga, S., Suzuki, K., Sugimoto, K., Kakizaki, Y. & Tanaka, H. (2000). Complete occulusion of left renal artery in pediatric-onset Takayasu's arteritis. *Tohoku Journal of Experimental Medicine, 190*(4), 289–294.
486. Toiviainen-Salo, S., Markula-Patjas, K., Kerttula, L., Soini, I., Valta, H. & Mäkitie, O. (2012). The thoracic and lumbar spine in severe juvenile idiopathic arthritis: Magnetic resonance imaging analysis in 50 children. *Journal of Pediatrics, 160*(1), 140–146. https://doi.org/10.1016/j.jpeds.2011.06.030
487. Tronconi, E., Miniaci, A., Baldazzi, M., Greco, L. & Pession, A. (2018). Biologic treatment for chronic recurrent multifocal osteomyelitis: report of four cases and review of the literature. *Rheumatology International, 38*(1), 153–160. https://doi.org/10.1007/s00296-017-3877-0
488. Tyrrell, P. N. M., Cassar-Pullicino, V. N., Eisenstein, S. M., Monach, J. F., Darby, A. J. & McCall, I. W. (1996). Back pain in childhood. *Annals of the Rheumatic Diseases, 55*(11), 789–793.
489. Vendhan, K., Sen, D., Fisher, C., Ioannou, Y. & Hall-Craggs, M. A. (2014). Inflammatory changes of the lumbar spine in children and adolescents with enthesitis-related arthritis: Magnetic resonance imaging findings. *Arthritis Care and Research, 66*(1), 40–46. https://doi.org/10.1002/acr.22201

Haematological and vascular diseases

490. Alonso Fernández, L., Nzau, M. & Ventureyra, E. (2008). Spinal intradural arteriovenous fistula with unusual presentation: Case report and literature review. *Child's Nervous System*, *24*(11), 1349–1353. https://doi.org/10.1007/s00381-008-0656-y
491. Brekeit, K. A. (2012). Successful repair of a contained rupture of mycotic aortic aneurysm in an 8-year-old child using polytetrafluoroethylene graft. *Saudi Medical Journal*, *33*(7), 787–790.

492. Chuang, N. A., Shroff, M. M., Willinsky, R. A., Drake, J. M., Dirks, P. B. & Armstrong, D. C. (2003). Slow-flow spinal epidural AVF with venous ectasias: two pediatric case reports. *AJNR Am J Neuroradiol*, *24*(9), 1901–1905.
493. Chun, J. Y., Gulati, M., Halbach, V. & Lawton, M. T. (2004). Thrombosis of a spinal arteriovenous malformation after hemorrhage: Case report. *Surgical Neurology*, *61*(1), 92–94. https://doi.org/10.1016/S0090-3019(03)00304-5
494. Griggs, J. R., Bricker, J. T., Mariscalco, M. M., Jefferson, L. S. & Langston, C. (1990). Back pain with cardiovascular collapse in a pediatric emergency department patient. *Pediatr Emerg Care*, *6*(1), 17–20.
495. Gupta, G., Singh, R., Kotasthane, D. S., v. d. Kotasthane & Kumar, S. (2010). Xanthogranulomatous Pyelonephritis in a male child with renal vein thrombus extending into the inferior vena cava: A Case Report. *BMC Pediatrics*, *10.* https://doi.org/10.1186/1471-2431-10-47
496. Nadig, M., Munshi, I., Short, M. P., Tonsgard, J. H., Sullivan, C. & Frim, D. M. (2000). A child with neurofibromatosis-1 and a lumbar epidural arteriovenous malformation. *J Child Neurol*, *15*(4), 273–275.
497. Nichols, J. L., Gonzalez, S. C., Bellino, P. J. & Bieber, E. J. (2010). Venous thrombosis and congenital absence of inferior vena cava in a patient with menorrhagia and pelvic pain. *J Pediatr Adolesc Gynecol*, *23*(1), e17-21. https://doi.org/10.1016/j.jpag.2009.04.007
498. Onur, O., Sivri, A., Gümrük, F. & Altay, C. (1999). Beta thalassaemia: A report of 20 children. *Clinical Rheumatology*, *18*(1), 42–44. https://doi.org/10.1007/s100670050050
499. Paine, R. S. & Efron, M. L. (1963). Atypical Variants of the 'Ataxia Telangiectasia' Syndrome: Report of Two Cases, Including One with Apparent Dominant Inheritance. *Developmental Medicine & Child Neurology*, *5*(1), 14–23. https://doi.org/10.1111/j.1469-8749.1963.tb04985.x
500. Petrov, I., Kaneva-Nencheva, A., Levunlieva, E., Genova, K., Garvanski, I., Konstantinov, G. & Adam, G. (2016). Successful endovascular treatment of type B aortic dissection in a 15-year-old child. *Cor et Vasa.* Scopus. https://doi.org/10.1016/j.crvasa.2016.04.006
501. Roger, E. & Letts, M. (1999). Sickle cell disease of the spine in children. *Can J Surg*, *42*(4), 289–292.
502. Saad, D. F., Gow, K. W., Redd, D., Rausbaum, G. & Wulkan, M. L. (2005). Renal artery pseudoaneurysm secondary to blunt trauma treated with microcoil embolization. *J Pediatr Surg*, *40*(11), e65-7. https://doi.org/10.1016/j.jpedsurg.2005.07.011
503. Tribe, H. & Borgstein, R. (2013). Dysgenesis of the inferior vena cava associated with deep venous thrombosis and a partial Protein C deficiency. *Journal of Radiology Case Reports*, *7*(11), 46–52. https://doi.org/10.3941/jrcr.v7i11.1485
504. Vogt, B. A., Birk, P. E., Panzarino, V., Hite, S. H. & Kashtan, C. E. (1999). Aortic dissection in young patients with chronic hypertension. *Am J Kidney Dis*, *33*(2), 374–378.
505. Ware, S. M., Shikany, A., Landis, B. J., James, J. F. & Hinton, R. B. (2014). Twins with progressive thoracic aortic aneurysm, recurrent dissection and ACTA2 mutation. *Pediatrics*, *134*(4), e1218-23. https://doi.org/10.1542/peds.2013-2503
506. Wei, H. Y., Chung, H. T., Wu, C. T. & Huang, J. L. (2011). Aortic dissection complicated with hemothorax in an adolescent patient with systemic lupus erythematosus: Case report and review of literature. *Semin Arthritis Rheum*, *41*(1), 12–18. https://doi.org/10.1016/j.semarthrit.2010.08.002
507. Yigit, H., Yagmurlu, B., Yigit, N., Fitoz, S. & Kosar, P. (2006). Low back pain as the initial symptom of inferior vena cava agenesis. *AJNR Am J Neuroradiol*, *27*(3), 593–595.
508. Zhang, H., He, M. & Mao, B. (2006). Thoracic spine extradural arteriovenous fistula: Case report and review of the literature. *Surg Neurol*, *66*(1), S18-23; discussion S23-4. https://doi.org/10.1016/j.surneu.2006.06.001

Abdominal and thoracic diseases

509. Alon, U. S. & Berenbom, A. (2000). Idiopathic hypercalciuria of childhood: 4- to 11-year outcome. *Pediatric Nephrology, 14*(10-11), 1011–1015.
510. Buick, R. G. & Chowdhary, S. K. (1999). Backache: A rare diagnosis and unusual complication. *Pediatric Surgery International, 15*(8), 586–587. https://doi.org/10.1007/s003830050680
511. Coscia, M. F., Hormuth, D. A. & Huang, W. L. (1992). Back pain secondary to esophageal perforation in an adolescent. *Spine, 17*(10), 1256–1259.
512. Dane, C., Dane, B., Erginbas, M. & Cetin, A. (2007). Imperforate hymen-a rare cause of abdominal pain: Two cases and review of the literature. *J Pediatr Adolesc Gynecol, 20*(4), 245–247. https://doi.org/10.1016/j.jpag.2006.12.003
513. Dasari, P. (2011). Torsion hematosalpinx and paraovarian cyst mimicking bilateral ovarian neoplasm in an adolescent girl. *Journal of Gynecologic Surgery, 27*(4), 285–287. https://doi.org/10.1089/gyn.2010.0056
514. Deathe, A. B. (1993). Hematometra as a cause of lumbar radiculopathy. A case report. *Spine (Phila Pa 1976), 18*(13), 1920–1921.
515. Deeg, K. H. & Mitsiouli, O. (2003). Hematometrocolpos: A rare cause of acute obstructive urinary retention in 3 pubertal girls. *Monatsschrift für Kinderheilkunde, 151*(7), 732–737. https://doi.org/10.1007/s00112-002-0483-2

516. Domany, E., Gilad, O., Shwarz, M., Vulfsons, S. & Garty, B. Z. (2013). Imperforate hymen presenting as chronic low back pain. *Pediatrics, 132*(3), e768-70. https://doi.org/10.1542/peds.2012-1040
517. Drakonaki, E. E.; Tritou, I.; Pitsoulis, G.; Psaras, K.; Sfakianaki, E. (2010). Hematocolpometra due to an imperforate hymen presenting with back pain: Sonographic diagnosis. Hematocolpometra due to an imperforate hymen presenting with back pain: sonographic diagnosis. E. *J Ultrasound Med., 29*(2, 321-322. doi: 10.7863/jum.2010.29.2.321
518. Fick, G. M., Duley, I. T., Johnson, A. M., Strain, J. D., Manco-Johnson, M. L. & Gabow, P. A. (1994). The spectrum of autosomal dominant polycystic kidney disease in children. *J Am Soc Nephrol, 4*(9), 1654–1660
519. Igarashi, T., Sekine, T., Sugimura, H., Hayakawa, H. & Arayama, T. (1993). Acute renal failure after exercise in a child with renal hypouricaemia. *Pediatric Nephrology, 7*(3), 292–293. https://doi.org/10.1007/BF00853226
520. Lee, Y. J. & Barker, R. (2016). An unusual cause of back pain in a child: Spinal subdural haematoma secondary to intracranial arachnoid cyst haemorrhage. *Quantitative Imaging in Medicine and Surgery, 6*(4), 478–481. https://doi.org/10.21037/qims.2016.08.02
521. Letts, M. & Haasbeek, J. (1990). Hematocolpos as a cause of back pain in premenarchal adolescents. *Journal of Pediatric Orthopaedics, 10*(6), 731–732.
522. Löllgen, R. M., Sabo, J., Mettler, A., Liniger, B. & Berger, S. (2016). Unique Presentation of Hematometrocolpos Mimicking Cauda Equina Syndrome: Severe Back Pain and Urinary Incontinence in an Adolescent Girl. *J Emerg Med, 51*(2), e19-23. https://doi.org/10.1016/j.jemermed.2016.01.031
523. Malley, M., Monaghan, M., Esmail, A., Neophytou, C. & Cheng, A. (2016). An unusual cause of back pain. *Arch Dis Child Educ Pract Ed, 101*(6), 316–318. https://doi.org/10.1136/archdischild-2015-308974
524. Odriozola Grijalba, M., Maduta, T., Villalobos Salguero, F. J., Congost Marín, S., Lalaguna Mallada, P., Vara Callau, M. & Perales Martínez, J. I. (2016). Low back pain in 12-year old adolescent as clinical manifestation of hematocolpos secondary to imperforate hymen. *Revista Espanola de Pediatria, 72*(1), 60–62.
525. Parkhad Suchitra, B., Palve Sachin, B., Latti, R. G. & Kulkarni, N. B. (2013). Effect of yoga on premenstrual and menstrual cycle disorders in adolescent girls. *Biomedicine (India), 33*(2), 170–175.
526. Wang, W., Chen, M. H., Yang, W. & Hwang, D. L. (2004). Imperforate hymen presenting with chronic constipation and lumbago: Report of one case. *Acta Paediatrica Taiwanica, 45*(6), 340–342.
527. Yekeler, E. & Ulutas, H. (2012). Bilateral chylothorax after severe vomiting in a child. *Ann Thorac Surg, 94*(1), e21-3. https://doi.org/10.1016/j.athoracsur.2012.01.023
